# Metformin induces Ferroptosis by inhibiting UFMylation of SLC7A11 in breast cancer

**DOI:** 10.1186/s13046-021-02012-7

**Published:** 2021-06-23

**Authors:** Jingjing Yang, Yulu Zhou, Shuduo Xie, Ji Wang, Zhaoqing Li, Lini Chen, Misha Mao, Cong Chen, Aihua Huang, Yongxia Chen, Xun Zhang, Noor Ul Hassan Khan, Linbo Wang, Jichun Zhou

**Affiliations:** 1 Department of Surgical Oncology, Sir Run Run Shaw Hospital, Zhejiang University, Hangzhou, 310000 Zhejiang China; 2 Biomedical Research Center and Key Laboratory of Biotherapy of Zhejiang Province, Hangzhou, 310000 Zhejiang China; 3 Department of Pathology, Sir Run Run Shaw Hospital, Zhejiang University, Hangzhou, 310000 Zhejiang China; 4 Zhejiang University School of Medicine, Hangzhou, 310031 Zhejiang China

**Keywords:** Metformin, Ferroptosis, UFMylation, SLC7A11, Breast cancer

## Abstract

**Background:**

Ferroptosis is a newly defined form of regulated cell death characterized by the iron-dependent accumulation of lipid peroxidation and is involved in various pathophysiological conditions, including cancer. Targeting ferroptosis is considered to be a novel anti-cancer strategy. The identification of FDA-approved drugs as ferroptosis inducers is proposed to be a new promising approach for cancer treatment. Despite a growing body of evidence indicating the potential efficacy of the anti-diabetic metformin as an anti-cancer agent, the exact mechanism underlying this efficacy has not yet been fully elucidated.

**Methods:**

The UFMylation of SLC7A11 is detected by immunoprecipitation and the expression of UFM1 and SLC7A11 in tumor tissues was detected by immunohistochemical staining. The level of ferroptosis is determined by the level of free iron, total/lipid Ros and GSH in the cells and the morphological changes of mitochondria are observed by transmission electron microscope. The mechanism in vivo was verified by in situ implantation tumor model in nude mice.

**Results:**

Metformin induces ferroptosis in an AMPK-independent manner to suppress tumor growth. Mechanistically, we demonstrate that metformin increases the intracellular Fe^2+^ and lipid ROS levels. Specifically, metformin reduces the protein stability of SLC7A11, which is a critical ferroptosis regulator, by inhibiting its UFMylation process. Furthermore, metformin combined with sulfasalazine, the system x_c_^−^ inhibitor, can work in a synergistic manner to induce ferroptosis and inhibit the proliferation of breast cancer cells.

**Conclusions:**

This study is the first to demonstrate that the ability of metformin to induce ferroptosis may be a novel mechanism underlying its anti-cancer effect. In addition, we identified SLC7A11 as a new UFMylation substrate and found that targeting the UFM1/SLC7A11 pathway could be a promising cancer treatment strategy.

**Supplementary Information:**

The online version contains supplementary material available at 10.1186/s13046-021-02012-7.

## Background

Due to its high efficacy, low cost, and safety, the biguanide metformin is the most widely used oral anti-diabetic drug. In addition, potential clinical applications other than diabetes have been proposed, such as cancer prevention and treatment [1], anti-aging [2], anti-haze [3] activity, and other applications. Among them, validation of metformin’s anti-tumor clinical efficacy and exploration of the underlying mechanism have become a hot spot in the field of cancer research. Over time, more and more novel anti-tumorigenic mechanisms of metformin are gradually being revealed.

Metformin might influence tumorigenesis both indirectly, through systemic reduction of insulin levels, and directly, via induction of energetic stress [1]. On the one hand, metformin has been shown to suppress tumorigenesis by altering systemic endocrine and metabolic states and thereby reducing serum glucose and insulin levels. On the other hand, accumulating evidence has shown that metformin acts directly on tumor cells and exerts its antitumor biological effects through AMPK-dependent and/or AMPK-independent signaling pathways. Specifically, metformin can activate AMP-activated protein kinase (AMPK) to inactivate mammalian target of rapamycin (mTOR), change intracellular reactive oxygen species (ROS) levels, and inhibit mitochondrial functions [4]. Metformin has also been shown to regulate iron homeostasis in cells, which may be linked to cell metabolism [5]. With the advancement of research, the mechanism by which metformin regulates apoptosis, autophagy, and other cell death pathways have gradually been revealed, but the dominant cell death mechanism by which metformin mediates tumor suppression is still not clear [6, 7]. One of the key challenges in cancer research is how to effectively kill cancer cells while leaving healthy cells intact. Dissecting the principal molecular mechanisms of metformin’s antitumor activities and the major forms of death mediated by metformin can help us to develop novel treatments.

To proliferate and progress, cancer cells show a higher iron requirement than healthy cells. The dependence of cancer cells on iron has implications in a number of cell death pathways, including ferroptosis, an iron-dependent form of cell death. Uniquely, both iron excess and iron depletion can be utilized in anticancer therapies. The absorption, transportation, storage and utilization of iron are mediated by the membrane protein including transferrin receptor 1 (TFR1), the ferrireductase activity of STEAP3, divalent metal transporter 1 (DMT1, also termed SLC11A2), and ferritin (an iron storage protein complex), all affect the sensitivity of ferroptosis [8, 9]. When iron is depleted, ferritinophagy can maintain iron homeostasis through autophagic degradation of the iron-storage protein ferritin. Ferritin is recruited by the specific cargo receptor NCOA4 to autophagosomes and undergoes lysosomal degradation to release free iron [10]. In addition, iron metabolism can be regulated by mitochondria [11, 12],which are the sole sites of heme synthesis and the major sites for iron–sulfur cluster (ISC) biogenesis [10]. Iron is an essential trace element for proper cell functioning. Exploring its physiological functions and mechanism of action may provide potential therapeutic directions against cancer and other diseases.

Their dependence on iron makes cancer cells more susceptible to iron-catalyzed necrosis, known as ferroptosis [8]. Ferroptosis was first proposed by Stockwell as a novel regulated cell death in 2012 [13]. Unlike autophagy and apoptosis, ferroptosis is defined as an iron-dependent and reactive oxygen species (ROS)-reliant cell death with characteristic cytological changes, including decreased or vanished mitochondria cristae, ruptured outer mitochondrial membranes, and condensed mitochondrial membranes [14]. Many diseases, including tumors, have been reported to be associated with ferroptosis, and targeting ferroptosis to eliminate tumors is increasingly becoming a promising new approach for tumor treatment [14]. The biological significance of ferroptosis is expanding rapidly by virtue of the discovery that GPX4 and system x_c_^−^ are crucial regulators of ferroptosis and by the use of ferrostatins to inhibit ferroptosis in diverse contexts. One of the key bottlenecks for protection from ferroptosis is the availability of GSH, which serves as a redox equivalent for GPXs, including GPX4. GSH synthesis depends on the availability of intracellular cysteine, which can be generated from cystine imported from the extracellular space via the sodium-independent cystine/glutamate antiporter system x_c_^−^.

System x_c_^−^, the cysteine/glutamate transporter, is a heterodimer consisting of a heavy chain (4F2, gene name *SLC3A2*) and a light chain (xCT, gene name *SLC7A11*), [15]. xCT is often aberrantly overexpressed in many cancers [16], making it a weak spot for overcoming cancer by induction of ferroptosis. Interestingly, several FDA-approved drugs have been identified to function via ferroptosis induction in different cancer entities. Additionally, ferroptosis-inducing compounds and mechanisms have been identified and are broadly categorized as system x_c_^−^ inhibitors (Erastin, Sulfasalazine, Sorafenib), glutathione depleters (FIN56), direct GPX4 inhibitors (RSL3), or as an iron scavenger (Deferoxamine) [17]. Among these, sulfasalazine (SAS) is commonly used for treatment of rheumatoid arthritis and has been previously identified as an inhibitor of SLC7A11 transporter activity [18]. In addition, *Slc7a11* KO mice are viable with no obvious phenotype [19]. Therefore, SLC7A11 likely represents a better therapeutic target for cancer treatment than GPX4 because inhibiting SLC7A11 would presumably cause less toxicity in patients than inhibiting GPX4. Considering this, we have primarily focused on Sulfasalazine (which inhibits SLC7A11) in our studies.

UFM1 represents a small subclass of ubiquitin proteins discovered in 2004 [20]. It is composed of 85 amino acids and has a molecular weight of 9.1 kD. UFM1 has a tertiary structure similar to the ubiquitin molecule, but only 16% of its amino acid sequence is similar, and it is located in the nucleus and cytoplasm [21]. Similar to ubiquitin proteins, UFM1 can covalently bind to target proteins through a series of enzymatic cascade reactions: UBA5 activates UFM1 as an E1 enzyme, UFC1 acts as a UFM1 E2 binding enzyme, and UFL1 acts as a UFM1 E3 ligase [22, 23]. This series of reactions is called UFMylation. Recent studies have demonstrated that as a novel posttranslational modification, UFMylation is involved in the occurrence and development of various diseases, including breast cancer [24–26]. Here, we found that UFM1 can act as a target protein for metformin, and that UFM1 can modify SLC7A11, which can thus be regulated by metformin.

Fully understanding the regulation mechanism of SLC7A11 is helpful to find an effective target for cancer therapy. Previously, the tumor suppressors p53 [27], BAP1 [28], and NRF2 [29] have been identified as upstream regulators of SLC7A11 which play a significant role in ferroptosis. In particular, recent studies have shown that NRF2 can regulate metabolic pathways including glutamine metabolism [30] and oxidative stress [31] that are associated with ferroptosis. And since metformin can regulate the expression of p53 [32] and NRF2 [33], the role of these genes in the regulation of ferroptosis by metformin could be a direction for further research in the future.

In this study, for the first time, we describe the involvement of ferroptosis in metformin-induced cell death and tumor inhibition, as well as the mechanism of metformin in regulating ferroptosis, and evaluate the strategies of metformin combined with ferroptosis inducers (Sulfasalazine) in the treatment of cancer. In short, our study demonstrates the function and mechanism of metformin in regulating ferroptosis in cancer cells through SLC7A11 UFMylation.

## Methods

### Cell culture

All cells were cultured in a 37 °C incubator in an atmosphere of 5% CO_2_. The human breast cancer cell lines (MCF-7, T47D, HCC1937, Bcap37, NHFB, HBL-100) were cultured in RPMI-1640 medium supplemented with 10% fetal bovine serum and 10,000 U/mL penicillin-streptomycin. The MDAMB231 and BT549 cell lines were cultured in Dulbecco’s modified Eagle’s medium supplemented with 10% fetal bovine serum and 10,000 U/mL penicillin-streptomycin. The cell culture media and supplements were purchased from Thermo Fisher Scientific.

### Reagents

Erastin (#HY15763), Sulfasalazine (#HY-14655), Z-VAD-FMK (#HY16658B), Ferrostatin-1(#HY100579), E-necrosulfonamide(#HY-100573), Cycloheximide (#HY-12320), 3-Methyladenine(#HY19312), Deferoxamine mesylate (#HYB0988), and AICAR(#HY-13417), Compound C (#HY-13418A) were purchased form MedChemExpress (MCE). 

### Western blotting

For the Western blotting analysis, cell lysates were collected by addition of lysis buffer supplemented with protease and phosphatase inhibitors for 30 min on ice. The cell lysates were centrifuged at 12,000 rpm for 15 min at 4 °C, and the supernatants were collected and quantified using the Bradford method. Between 20 and 50 μg of proteins were diluted in 5× SDS loading buffer, boiled at 100 °C for 10 min, separated on SDS-PAGE gels and then transferred to polyvinylidene fluoride membrane. The membranes were blocked with 5% milk in 0.1% Tween-TBS at room temperature for 1 h and then incubated with the primary antibodies at 4 °C overnight. The following antibodies were used: SLC7A11 (Abcam, ab37185), UFM1 (Abcam, ab109305), GPX4 (Abcam, ab125066), 4-HNE (Abcam, ab46545), AMPK⍺-antibody (CST, #2532), Phospho-AMPK⍺-antibody (CST, #2535), GAPDH (Santa Cruz, sc-47,724), and HA (Santa Cruz, sc-7392). The corresponding HRP-conjugated secondary antibodies were incubated for 1 h at room temperature. Immunoreactive bands were detected by an ECL system (Amersham Biosciences) using an image reader. Densitometric analysis was performed by ImageJ. The data were then corrected by background subtraction and normalized against GAPDH as an internal control.

### qRT-PCR

Total RNA was extracted using TRIzol reagent, and cDNA was synthesized with SuperScript II Reverse Transcriptase. Quantitative real-time PCR was performed using SYBR GreenER qPCR SuperMix Universal, and triplicate samples were run on a Stratagene MX3000P qPCR system according to the manufacturer’s protocol. The threshold cycle (Ct) values for each gene were normalized to those of GAPDH, and the 2 − ΔΔCt method was used for quantitative analysis. The following primers were used:

- Q-SLC7A11-F: TCATTGGAGCAGGAATCTTCA
- Q-SLC7A11-R: TTCAGCATAAGACAAAGCTCCA
- Q-UFM1-F: CAGTGTTCCTGAAAGTACACCTT
- Q-UFM1-R: CCGCAGTTCTGAACCATGTTTTA
- Q-GAPDH-F: TGTGGGCATCAATGGATTTGG
- Q-GAPDH-R: ACACCATGTATTCCGGGTCAAT

### siRNA plasmid and transfection

For in vitro assays, knockdown of SLC7A11 and UFM1 proteins was performed by transfection of cells with siRNA duplex oligos using Lipofectamine 3000 (Invitrogen, no.2189668) in OPTI-MEM (Gibco, no.2185849) overnight. The corresponding siRNA sequence were as follows:

- siSLC7A11#1: UGGAGUUAUGCAGCUAAUU
- siSLC7A11#2: GAGGUCAUUACACAUAUAU
- siUFM1#1: GUUGGAAGUUGUUAAUAUC
- siUFM1#2: GAACUGCGGAUUAUUCCUA

### Colony formation assay

For the colony formation assay, 1000 cells were seeded in 6-well plates. Cells were treated with DMSO or Metformin. After 10–14 days of incubation, colonies were fixed with 4% paraformaldehyde for 20 min and stained with 0.5% crystal violet for 10 min. The crystal violet was carefully removed, and the cells were rinsed with tap water. The plates with colonies were allowed to dry in normal air at room temperature. Plating efficiency was determined for each cell line, and the surviving fraction was calculated based on the number of colonies that arose after treatment.

### Cell viability assay

Cell viability was evaluated using Cell Counting Kit-8 (CCK-8). Cells were seeded in a 96-well plate, and after cell treatments, CCK-8 reagent was added according to the manufacturer’s instructions. Absorbance was measured at 450 nM using a standard instrument.

### Cell death assay

Propidium iodide (PI, Invitrogen) was used as a fluorescent signal for cell death, and images were taken after metformin treatment with or without DFO and Fer-1. For PI imaging, the cultures were supplemented with 5 mg/ml PI. After a 30 min incubation, brightfield and PI images were acquired. Quantitative analysis of cell death was measured by PI staining coupled with flow cytometry.

### Total ROS assay

The total ROS level in cells was assessed using a Reactive oxygen species detection kit (Beyotime, S0033S). Cells were treated as indicated, and then, 10 μmol/L DCFH-DA diluted in serum-free medium was added and incubated with the cells at 37 °C for 20 min. Excess DCFH-DA was removed by washing the cells three times with serum-free cell culture medium. DCFH-DA can be hydrolyzed by intracellular esterases to produce DCFH. Labeled cells were trypsinized and resuspended in serum-free medium. Intracellular reactive oxygen species can oxidize nonfluorescent DCFH to produce DCF, which is fluorescent. The fluorescence of DCF was analyzed using flow cytometry.

### Lipid ROS assay

The relative lipid ROS level in cells was assessed using C11-BODIPY dye (Thermo Fisher Scientific, D3861). Cells were treated with 5 μm C11-BODIPY for 30 min, harvested, washed twice with PBS and resuspended in 500 μl PBS. Oxidation of the polyunsaturated butadienyl portion of the dye results in a shift of the fluorescence emission peak from ~ 590 nm to ~ 510 nm.

### GSH assay

The relative GSH concentration in cell lysates was assessed using a total Glutathione Assay Kit (Beyotime, S0052) according to the manufacturer’s instructions. Briefly, Glutathione reductase reduces oxidized glutathione (GSSG) to reduced glutathione (GSH), which reacts with the chromatin substrate DTNB to produce yellow TNB and GSSG. The combination of the two reactions reveals the total glutathione (GSSG + GSH), and the amount of TNB (yellow) formation represents the amount of total glutathione. Thus, the total glutathione content can be calculated by measuring A412.

### Iron assay

Intracellular chelatable iron was determined using the fluorescent indicator Phen Green SK from Thermo Fisher Scientific (P-14313). PSK-green was diluted with HBSS to 5 μM. Cells were washed twice with HBSS and incubated with 5 μM PSK-green at 37 degrees for 30 min. Then, the cells were washed with HBSS, digested with trypsin and collected by centrifugation. The fluorescence of Phen Green SK was detected using flow cytometry.

### Transmission electron microscopy

Cells cultured in a 6-well plate were fixed with a solution containing 2.5% glutaraldehyde in PBS for 24 h. After being washed in 0.1 M PBS, the cells were treated with 0.1% Millipore-filtered cacodylate-buffered tannic acid, postfixed with 1% buffered osmium, and stained with 1% Millipore-filtered uranyl acetate. After dehydration and embedding, samples were incubated in a 60 °C oven for 24 h. Digital images were obtained using a transmission electron microscope. Mitochondrial density is Quantitative analysis by the ImageJ software. Cell Number ≥ 3, mitochondria Number ≥ 17, *p < 0.05, t-test.

### Protein stability assay

Cells were treated with 100 μg/mL cycloheximide (CHX) for a variety of time intervals after knockdown of UFM1. Then SLC7A11 protein lysate was resolved on SDS-PAGE and analyzed by western blotting.

### UFMylation modification detection

UFM1 is mainly bound to its target protein through covalent bonding. Therefore, to detect whether a protein can be modified by UFM1, the interference of noncovalent binding needs to be eliminated. Cells were lysed with a strong denaturant containing SDS and then boiled at 100 °C for 10 min to remove noncovalently bound UFM1 protein. Immunoprecipitation was performed with the supernatant.

### Immunohistochemistry and digital pathology analysis

Tissue sections from the indicated mouse models were fixed in 10% buffered formalin and embedded in paraffin. For IHC staining, tissue slides were deparaffinized in xylene and rehydrated in alcohol. Endogenous peroxidase was blocked with 3% hydrogen peroxide. Antigen retrieval was achieved using a microwave and 0.1 M citric sodium buffer (pH 6.0). Sections were then incubated overnight at 4 °C with the primary antibody. Then three PBS washes were performed, after which the slides were exposed to the secondary antibody (ZGBBT, PV-9001) for 1 h at room temperature. We wash 3 times with PBS, 5 min each time, then with DAB color development for 5 min, tap water for 10 min, hematoxylin counterstain for 2 min, tap water again for 10 min, and then routine dehydration, transparency, mounting, and microscopy. And for each staining, a positive control was included (human breast cancer tissues), as well as a negative control, without the primary antibody or with rabbit/ mouse IgG.

Immunostained slides of each histology sample from serially cut tumor sections were scanned at magnification × 20. The SLC7A11 and 4-HNE were localized on the cell membrane and cytoplasm respectively. The UFM1 was localized on the cell membrane and cytoplasm. The specimens were evaluated according to the intensity of staining (no staining = 0, weak staining = 1, moderate staining = 2, strong staining = 3) and the extent of stained cells (0% = score 0, 1–10% = 1; 11–50% = 2; > 51% = score3). Negative means 0% area staining. Focally positive means 1–80% area staining, diffusely positive means 81–100% area staining. Values were expressed as mean ± SD.

### Xenograft studies

Animal studies were reviewed and approved by the Ethics Committee for Animal Studies of Zhejiang University. T47D xenografts were established in 5- week-old nude mice (Shanghai SLAC Laboratory Animal Corporation) by inoculating 1 × 10^7^ cells mixed with Matrigel (BD Biosciences) at 1:1 ratio (volume) into the abdominal mammary fat pad. When the tumor reached 50–100 mm^3^, the mice were assigned randomly into different treatment groups (DMSO, Metformin, SAS, and Metformin + SAS groups). Metformin (200 mg/kg/day) was provided in drinking water. Sulfasalazine was dissolved in dimethyl sulfoxide (DMSO), diluted in PBS, and then intraperitoneally injected into mice at a dose of 250 mg/kg once a day. Tumor sizes were measured every 3 days, and tumor volumes were calculated as follows: length × width^2^ × 0.5. Each group consisted of 5 mice. After 21 days of treatment, all mice were euthanized, and the tumors were surgically removed. A portion of the tumors was immediately fixed in 10% buffered formalin for immunohistochemistry.

### Bioinformatics

Kaplan Meier plot showing influence of SLC7A11 or UFM1 expression level on overall survival of BRCA patients. We identified the upper and lower cut-off values using:75 and 25%. Statistical tests and *P*-values were two-sided. Differences were considered significant with a value of *P* < 0.05.

### Software and statistical analysis

Flow cytometry data were analyzed using Flow Jo software (X.10.0.7r2). The schematic diagram was drawn using ChemDraw Ultra (16.0.1.4). Statistical analyses were carried out using Microsoft Excel software and GraphPad Prism to assess differences between experimental groups. Pearson’s correlation (two-sided) was performed to analyse gene correlation of UFM1 and SLC7A11 expression in different cancer types. Data are presented as mean ± SD from 3 independent experiments. P Statistical significance was determined using a two-tailed, unpaired Student’s t-test with a confidence interval of 95%. *P* ≤ 0.05 was considered statistically significant (*P < 0.05; **P < 0.01; ***P < 0.001; ****P < 0.0001).

## Results

### Metformin triggers Iron-dependent cell death

**Fig. 1:**
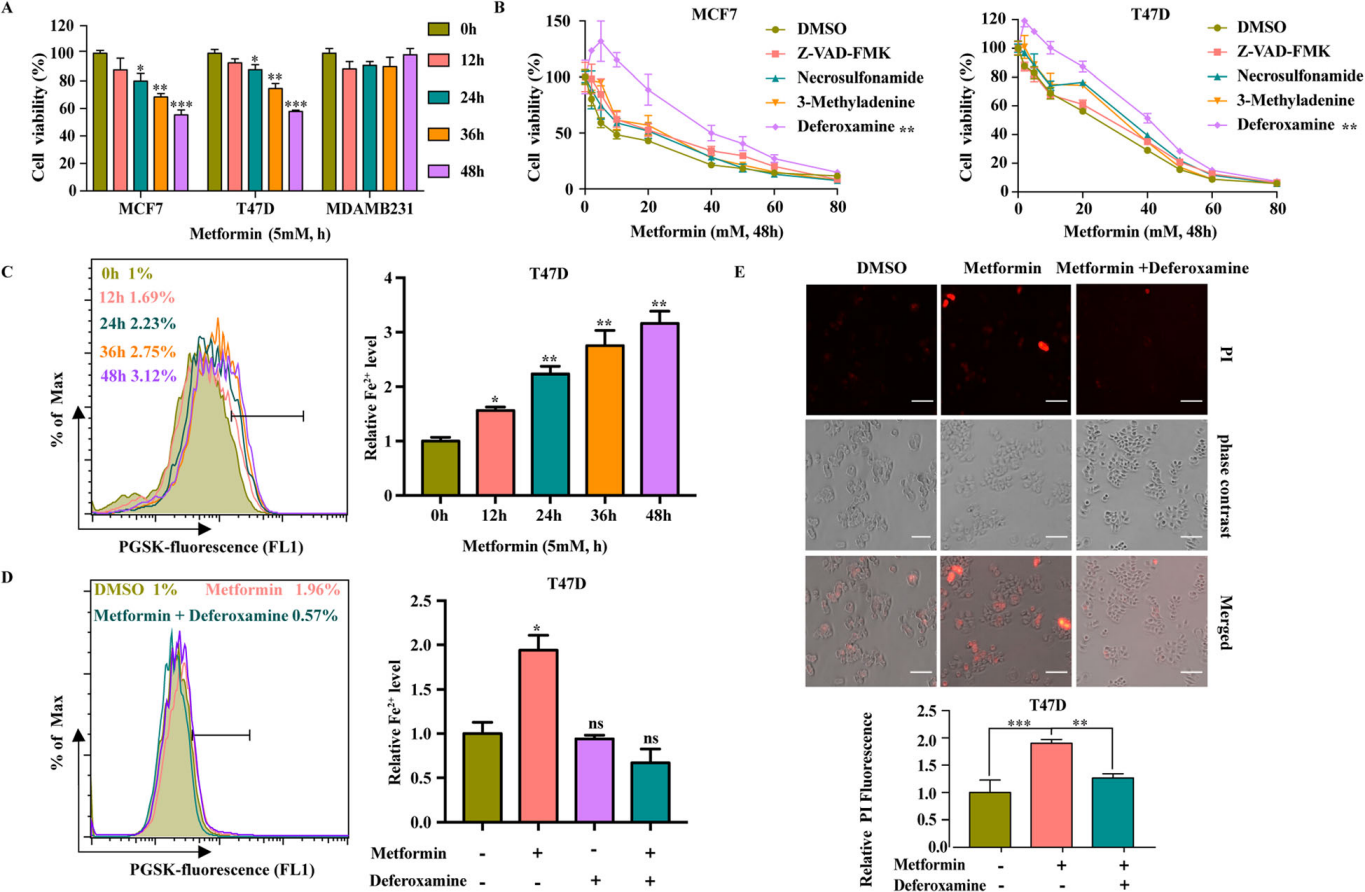
Metformin-Induced Cell Death can be Inhibited by DFO. **A** Breast cancer lines were treated with metformin (5 mM) for 0, 12, 24, 36, or 48 h, and cell viability was assayed. **B** MCF7 and T47D cells were treated with metformin (0–80 mM) in the absence or presence of Z-VAD-FMK (20 μM), necrosulfonamide (1 μM), 3-Methyladenine (5 mM) or Deferoxamine (20 μM) for 48 h, and cell viability was assayed. **C** T47D cells were treated with metformin (5 mM) for 0, 12, 24, 36, or 48 h, and the relative levels of Fe^**2+**^ were assayed. **D** T47D cells were treated with metformin (5 mM) for 48 h in the absence or presence of Deferoxamine. The relative levels of Fe^**2+**^ were assayed. **E** T47D cells were treated with metformin (5 mM) for 48 h in the absence or presence of Deferoxamine. Microscopy showing cell death. Propidium iodide (PI) staining indicates dead cells (scale bar, 100 mM). Quantitative analysis of cell death was measured by PI staining coupled with flow cytometry

Accumulating evidence has demonstrated that metformin has great potential in prevention and treatment of breast cancer in clinical settings, and metformin can effectively inhibit the proliferation of breast cancer [34, 35]. Consistently, our study also showed that metformin could effectively inhibit the proliferation of some breast cancer cells in dose- and time-dependent manner (Fig. 1a, Fig. S1A and B). Next, we explored whether these well-established forms of regulated cell death are involved in the anti-cancer activity of metformin in breast cancer cells. We used various cell death inhibitors, including Z-VAD-FMK (an apoptosis inhibitor), necrosulfonamide (a necroptosis inhibitor), and 3-Methyladenine (3-MA, an autophagy inhibitor). Since metformin has been shown to regulate iron homeostasis in cells, which may be linked to cell metabolism [5], we also introduced an iron scavenger, Deferoxamine (DFO) [36]. The results showed that DFO restored cell viability in T47D and MCF7 cells cultured with metformin (Fig. 1b). In our study, Z-VAD-FMK, necrosulfonamide, and 3-MA also slightly improved cell viability compared with DMSO (Fig. 1b). Although metformin can induce apoptosis and inhibit autophagy (Fig. S2A-D), the effect of DFO was much more significant than that of the apoptosis and autophagy inhibitors (Fig. 1b). Moreover, metformin promoted the accumulation of iron ions, and the increase in the iron ion level was inhibited by DFO (Fig. 1c and d). Propidium iodide staining confirmed that DFO inhibited metformin-induced cell death in T47D cells (Fig. 1e). Collectively, these findings indicate that iron ions are required for the anti-cancer effect of metformin, and that the increase in iron ions induced by metformin may be a mechanism by which metformin exerts its anti-cancer effect.

### Metformin can induce ferroptosis

Ferroptosis is an iron-dependent form of cell death [23]. Metformin can promote accumulation of iron ions, and DFO, as a ferroptosis inhibitor, can effectively inhibit the anti-cancer effect of metformin (Fig. 1c and d). Therefore, we hypothesized that the anti-cancer effect could be at least partly explained by metformin-mediated iron-dependent ferroptosis.

**Fig. 2:**
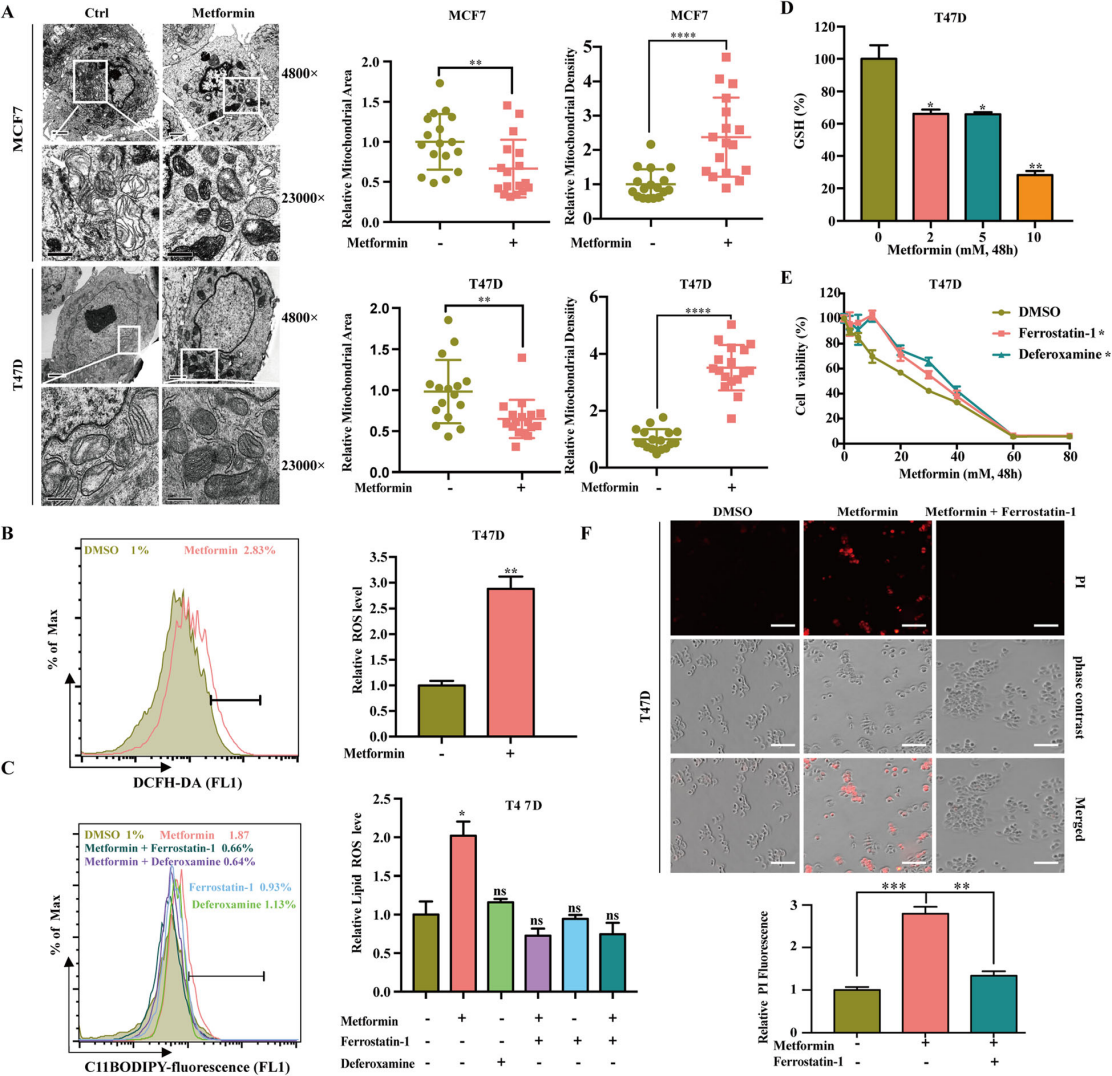
Metformin can Trigger ferroptosis. **A** Cell morphology was observed via transmission electron microscopy after cells were treated with metformin (5 mM) for 48 h. The area and density of mitochondrial is quantitative analysis by using the ImageJ software. **B** T47D cells were treated with metformin (5 mM) for 48 h. The relative total ROS levels were assayed via DCFH-DA fluorescence. **C** T47D cells were treated with metformin (5 mM) for 48 h in the absence or presence of Ferrostatin-1 (1 μM) and Deferoxamine (20 μM) for 48 h. The relative lipid ROS levels were assayed via C11-BODIPY fluorescence. **D** T47D cells were treated with metformin at 0, 2, 5, or 10 mM for 48 h, and the relative levels of GSH were assayed. **E** T47D cells were treated with metformin (0–80 mM) for 48 h in the absence or presence of Ferrostatin-1 (1 μM) and Deferoxamine (20 μM) for 48 h. Cell viability was assayed. **F** T47D cells were treated with metformin (5 mM) for 48 h in the absence or presence of Ferrostatin-1 (1 μM) for 48 h. Microscopy showing cell death. Propidium iodide (PI) staining indicates dead cells (scale bar, 100 mM). Quantitative analysis of cell death was measured by PI staining coupled with flow cytometry

To clarify the association between metformin and ferroptosis, we first investigated the effects of metformin on the morphological characteristics of ferroptosis. One of the unique morphological characteristics of ferroptosis is a decrease in mitochondrial volume and an increase in membrane density [37]. Transmission electron microscopy showed that metformin induced a decrease in mitochondrial volume and an increase in membrane density in MCF7 and T47D cells (Fig. 2a). Next, we focused on the effects of metformin on the biochemical processes of ferroptosis, including accumulation of total ROS and lipid peroxidation, which can be inhibited by ferroptosis inhibitors (DFO, Fer-1), and a reduction in the intracellular GSH level [37]. Therefore, breast cancer cells were treated with metformin at different concentrations, and the corresponding kits were used to detect these indicators. Unsurprisingly, our results showed that metformin increased intracellular total ROS and lipid ROS levels, and the increase of lipid ROS was inhibited by ferroptosis inhibitors (DFO, Fer-1) while DFO and Fer-1 themselves had little effect on these (Fig. 2b and c and Fig. S1C). In addition, metformin reduced intracellular GSH levels (Fig. 2d and Fig. S1D). Moreover, we tested whether ferroptosis inhibitors (DFO, Fer-1) could reverse the anti-cancer effect of metformin. Our results demonstrated that both DFO and Fer-1 could significantly rescue cell viability when T47D cells were treated with metformin (Fig. 2e). Furthermore, propidium iodide staining confirmed that Fer-1 inhibited metformin-induced cell death in T47D cells (Fig. 2f). In summary, metformin can induce ferroptosis.

### Metformin inhibits SLC7A11 expression

**Fig. 3:**
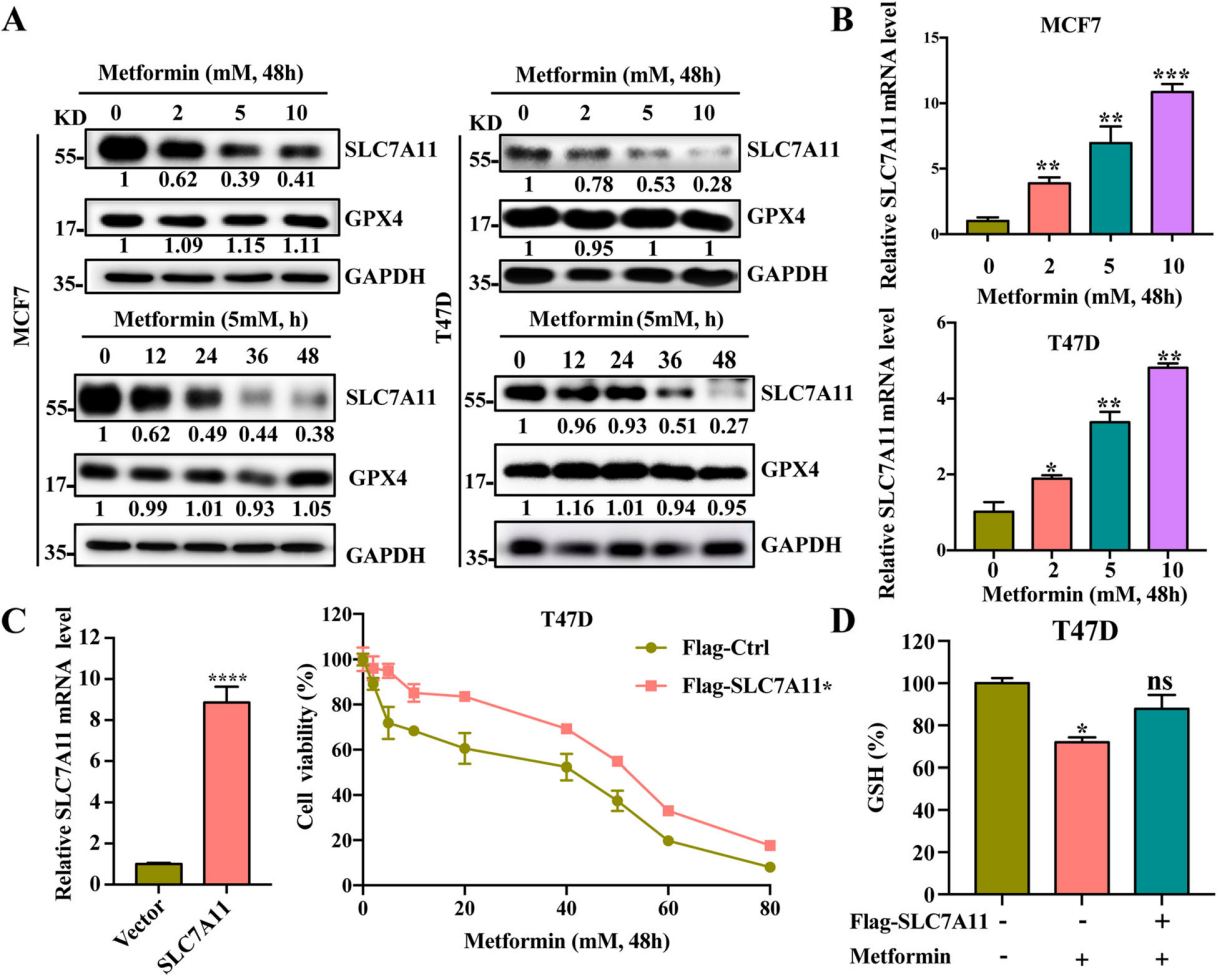
Metformin causes System X_c_^−^ dysfunction. **A** MCF7 and T47D cells were treated with metformin at 0, 2, 5, or 10 mM for 48 h or treated with metformin (5 mM) for 0, 12, 24, 36, or 48 h. SLC7A11 and GPX4 protein expression was measured via Western blotting. **B** The relative SLC7A11 RNA level was measured via qRT-PCR after MCF7 and T47D cells were treated with metformin at 0, 2, 5, or 10 mM for 48 h. **C** T47D cells were treated with metformin (0–80 mM) for 48 h with or without the overexpression of SLC7A11. Cell viability was assayed. **D** T47D cells were treated with 5 mM metformin for 48 h with or without the overexpression of SLC7A11. The relative levels of GSH were assayed

Next, we sought to study how metformin induced ferroptosis. Ferroptosis is triggered by lipid peroxidation and is tightly regulated by SLC7A11, a key component of the cystine-glutamate antiporter. GPX4 and GSH synergistically regulate lipid peroxidation [38, 39]. GSH generation is regulated by SLC7A11, which is a crucial factor in the glutamate transport system and plays a key role in the ferroptosis pathway [40, 41]. To identify the specific molecular mechanism of metformin in regulating ferroptosis, the effects of different metformin concentrations and treatment durations on the expression of SLC7A11 and GPX4 were detected. The results showed that metformin effectively inhibited the expression of SLC7A11 in T47D and MCF7 cells in a dose- and time-dependent manner while the level of transcription was significantly elevated (Fig. 3a and b). In addition, metformin had no significant effect on GPX4 expression (Fig. 3a). We also examined the inhibitory efficiency of metformin against SLC7A11 in other breast cancer cells. The results showed that metformin selectively inhibited the expression of some breast cancer cells (Fig. S3B). Therefore, metformin inhibits SLC7A11 expression in a posttranscriptional manner and may play its anti-cancer role by inhibiting the SLC7A11 protein level (Fig. S1A and B, Fig. S3B).

To further clarify the role of SLC7A11 in the anti-cancer effect of metformin, SLC7A11 was knocked down or overexpressed in T47D cells to detect changes in metformin sensitivity and its effect on lipid ROS and GSH levels. The results showed that knockdown of SLC7A11 significantly increased the sensitivity of cells to metformin and enhanced the production of Lipid ROS induced by metformin (Fig. S3C and S4C). And overexpression of SLC7A11 significantly reversed the anti-cancer effect of metformin and the upregulation of GSH (Fig. 3c and d and Fig. S3C). In addition, overexpression of SLC7A11 blocked the lipid ROS increase induced by metformin (Fig. S3D). Moreover, knock down or over-expression of SLC7A11 had no significant effect on cell proliferation (Fig. S4A). Therefore, we conclude that the effect of SLC7A11 in the inhibition of tumor cells by metformin may rule out the effect of SLC7A11 itself on cell proliferation. These results suggest that metformin can induce ferroptosis to suppress cancer by downregulating the SLC7A11 protein level.

### Metformin can regulate UFM1 expression

**Fig. 4:**
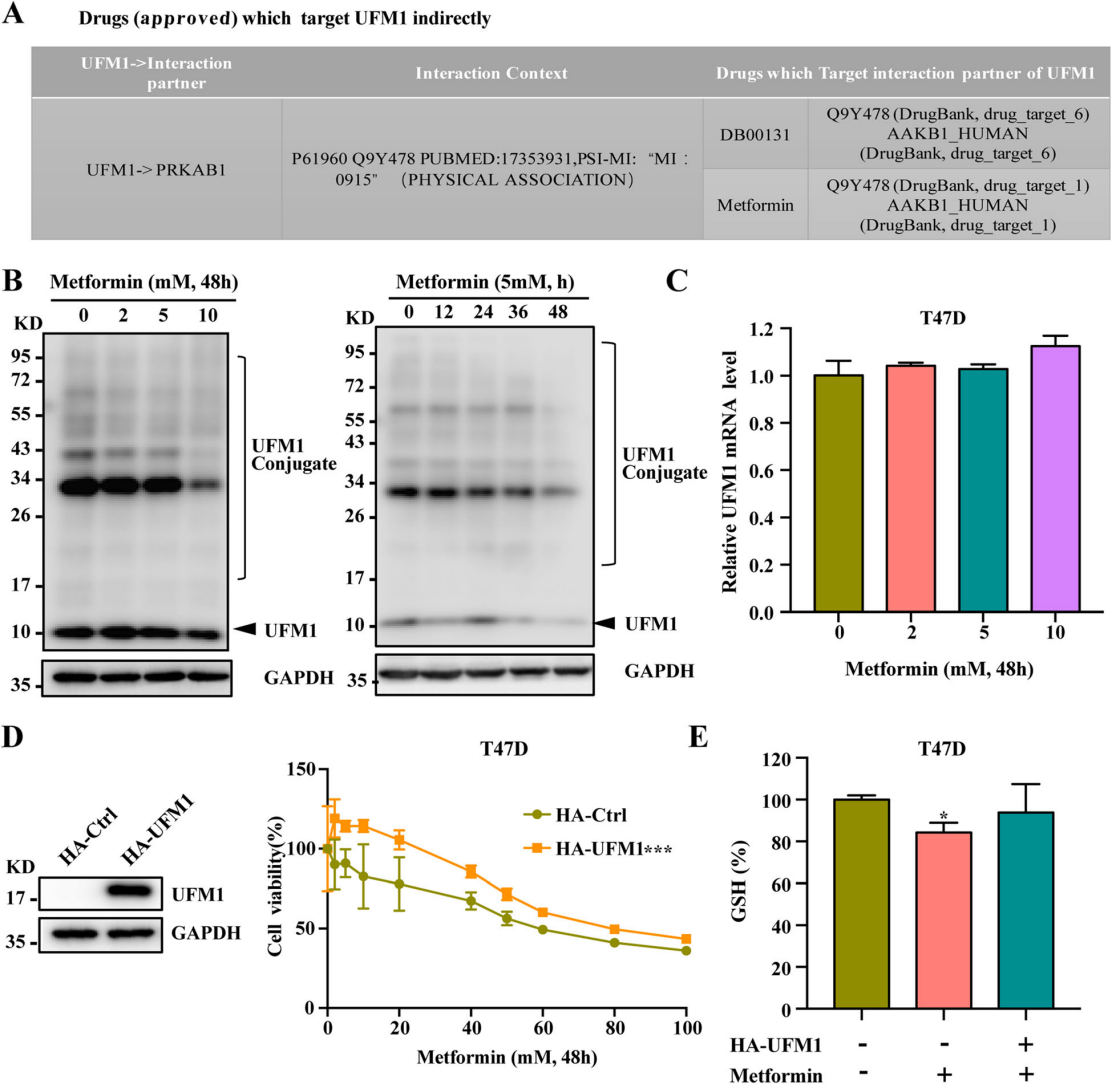
Metformin can regulate UFM1 expression. **A** DRUGSURV database (http://www.bioprofiling.de/GEO/DRUGSURV/) analysis showing that UFM1 is an indirect target of metformin. **B** T47D cells were treated with metformin at 0, 2, 5, or10 mM for 48 h or treated with metformin (5 mM) for 0, 12, 24, 36, or 48 h. The UFM1-conjugated proteins were measured via Western blotting. **C** The relative UFM1 RNA level was measured via qRT-PCR after T47D cells were treated with metformin at 0, 2, 5, or 10 mM for 48 h. **D** T47D cells were treated with Metformin (0–80 mM) for 48 h with or without the overexpression of UFM1. Cell viability was assayed. **E** T47D cells were treated with 5 mM metformin for 48 h with or without overexpression of UFM1. The relative levels of GSH were assayed

UFMylation is a ubiquitin-like modification that plays an important role in the occurrence and development of breast cancer. The UFM1 system is conserved in metazoans and plants but not in yeast, suggesting its specific roles in multicellular organisms [20]. Based on DRUGSURV database analysis (DRUGSURV: a resource for repositioning of approved and experimental drugs in oncology based on patient survival information), we found that UFM1 can be targeted by metformin indirectly (Fig. 4a). We speculated that UFM1 might play a certain role in the anti-cancer effect of metformin [24]. To detect the regulatory effect of metformin on UFM1, the effect of metformin on UFM1 expression was tested. The results showed that metformin inhibited the level of UFM1 protein and the overall UFMylation modification level (Fig. 4b) to some extent without affecting the UFM1 transcription level (Fig. 4c).

To further clarify the effect of UFM1 on the tumor inhibition activity of metformin, UFM1 was overexpressed in T47D cells. The results showed that overexpression of UFM1 effectively blocked the anti-cancer effect of metformin and restored the inhibition of the GSH level mediated by metformin (Fig. 4d and e). In addition, in order to rule out the effects of UFM1 on cell viability, we examined the effects of UFM1 on cell proliferation, the result show that over-expression of UFM1 had no significant effect on cell proliferation (Fig. S4B). Moreover, overexpression of UFM1 also blocked the lipid ROS induction effect of metformin (Fig. S3E). In summary, our results showed that metformin might exert its anti-cancer effect through UFM1, which can regulate the GSH level.

### Metformin downregulated SLC7A11 expression by inhibiting UFMylation of SLC7A11

**Fig. 5:**
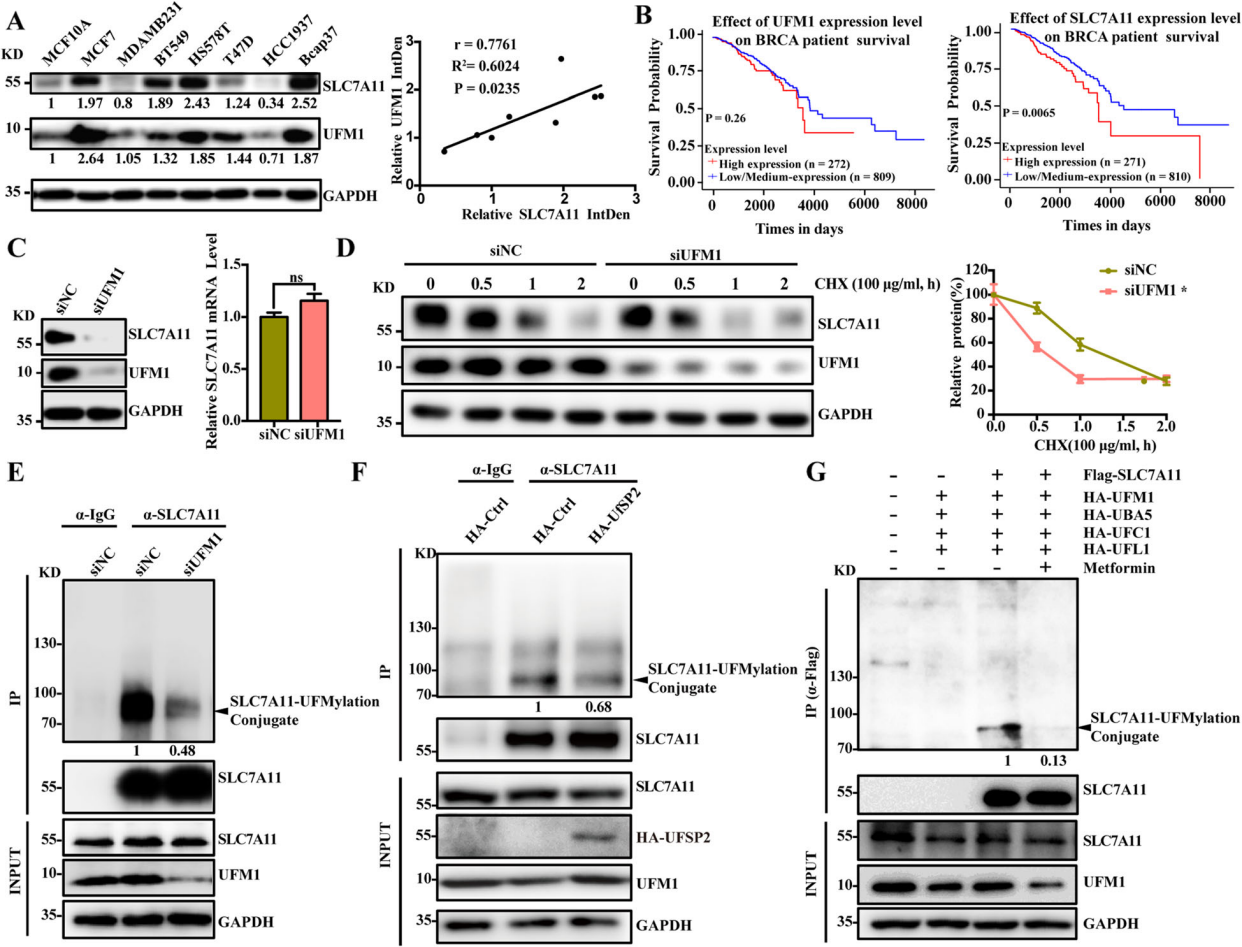
Metformin downregulated SLC7A11 expression by inhibiting UFMylation of SLC7A11. **A** UFM1 and SLC7A11 expression in breast cancer cell lines was detected via Western blotting. Scatter plots showing positive correlation of UFM1 and SLC7A11 expression in different cancer types. **B** TCGA database analyse the effect of UFM1 or SLC7A11 on survival. **C** After knockdown of UFM1 with siRNA, the relative SLC7A11 protein and RNA expression levels were measured via Western blotting and qRT-PCR. **D** The protein level of SLC7A11 in breast cancer cell lines was detected by Western blotting after cells were treated with cycloheximide (CHX, 100 μg/ml) after knockdown of UFM1. **E** After siRNA-mediated knockdown of UFM1, SLC7A11 UFMylation was measured via Co-IP. **F** SLC7A11 UFMylation was measured via Co-IP in the absence or presence of UfSP2. **G** SLC7A11 UFMylation was measured via Co-IP in the absence or presence of metformin

Ferroptosis is triggered by lipid peroxidation and is tightly regulated by SLC7A11, a key component of the cystine-glutamate antiporter [42], and thus, exploring its regulatory mechanism is helpful in finding further novel therapeutic targets for inducing ferroptosis in cancer therapy. Our results demonstrated that the ferroptosis inducer erastin downregulated the expression of UFM1 and SLC7A11, indicating that UFM1 may participate in ferroptosis regulation, and further confirming the correlation between SLCA7A11 and UFM1 (Fig. S3A). To clarify the role of UFM1 in ferroptosis regulation by metformin, we first detected the expression of SLC7A11 and UFM1 in different breast cancer cell lines. Our results showed that there was an obvious positive correlation between the SLC7A11 and UFM1 protein levels (Fig. 5a). Next, utilizing publicly available databases, our bioinformatics analysis showed that there was a significant negative correlation between the expression of SLC7A11 and the prognosis of breast cancer patients, but not UFM1(Fig. 5b).

To determine whether SLC7A11 and UFM1 have regulatory effects, the effect of SLC7A11 expression was detected after knockdown of UFM1. The results showed that knockdown of UFM1 downregulated the expression of SLC7A11 without affecting its transcription level (Fig. 5c). Furthermore, knockdown of UFM1 inhibited the protein stability of SLC7A11 (Fig. 5d).

To determine whether SLC7A11 could be modified by UFM1, UFMylation of SLC7A11 was detected with or without UFM1 knockdown. SLC7A11 was found to be a substrate for UFMylation, and the specificity of its modification was determined by knockdown of UFM1 (Fig. 5e). Covalent binding of UFM1 to a target protein can be reversed by the UfSP2 enzyme. Further tests of UFMylation modification showed that SLC7A11 UFMylation could be inhibited by UfSP2 (Fig. 5f). These findings define SLC7A11 as a new modification substrate of UFM1.

Furthermore, to clarify the specific molecular mechanism by which metformin regulates SLC7A11, we used metformin-treated cells to detect the effect of metformin on the level of SLC7A11 UFMylation. The results showed that metformin effectively inhibited the level of SLC7A11 UFMylation (Fig. 5g).

In summary, metformin downregulates SLC7A11 protein stability to induce ferroptosis by inhibiting UFMylation of SLC7A11.

### Metformin can induce ferroptosis independent of the AMPK pathway

**Fig. 6:**
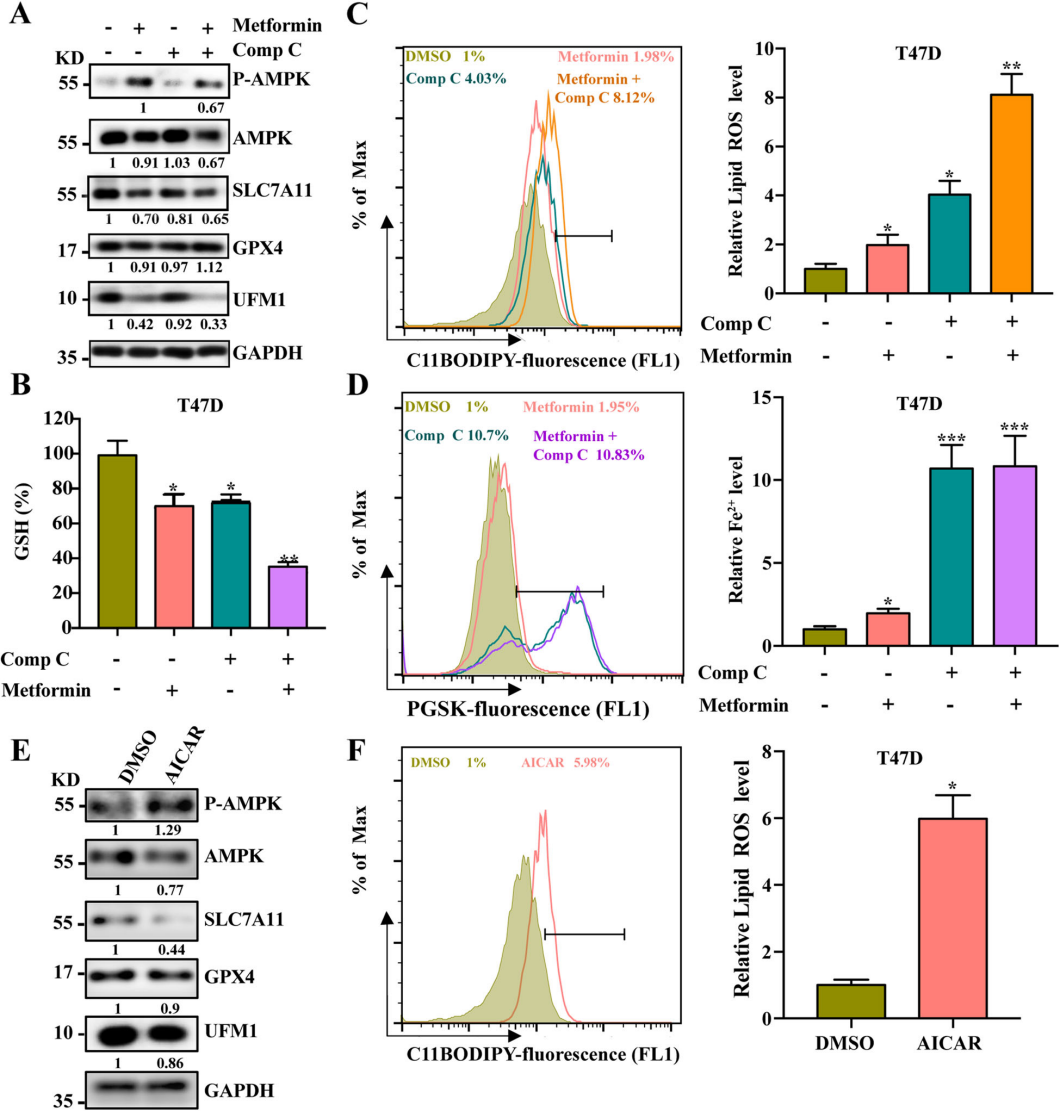
Metformin can induce ferroptosis independent of the AMPK pathway. **A** T47D cells were treated with metformin (5 mM) for 48 h in the absence or presence of Comp C (1 μM) for 48 h. Protein expression was measured via Western blotting. **B** T47D cells were treated with metformin (5 mM) for 48 h in the absence or presence of Comp C (1 μM) for 48 h, and the relative GSH levels were assayed. **C** and **D** T47D cells were treated with metformin (5 mM) for 48 h in the absence or presence of Comp C (1 μM) for 48 h. The relative lipid ROS and Fe^2+^ levels were assayed via C11-BODIPY and PSGK fluorescence, respectively. **E** T47D cells were treated with AICAR (2 mM) for 48 h, and protein expression was measured via Western blotting. **F** T47D cells were treated with AICAR (2 mM) for 48 h. The relative lipid ROS levels were assayed via C11-BODIPY fluorescence

At present, many studies have demonstrated that metformin can directly act on tumor cells and exerts its antitumor biological effects through AMPK-dependent and/or AMPK-independent signaling pathways [43]. Our results also show that metformin can activate AMPK phosphorylation (Fig. 6a). Therefore, to determine whether the AMPK pathway is involved in the regulation of metformin on ferroptosis, we firstly clarify whether the activation of AMPK can induce ferroptosis. The results show AICAR, an AMPK activator, increased lipid ROS production and inhibited SLC7A11 expression which indicated that AMPK activation can induce ferroptosis (Fig. 6e and f). To further determine the role of AMPK pathway in the ferroptosis induced by metformin, we treat cells with metformin in the absence or presence of Compound C, an inhibitor of AMPK. Interestingly, the results showed that the combination of metformin and Compound C can synergistically increase the level of Lipid ROS and inhibit the level of GSH and the expression of SLC7A11(Fig. 6a, b, c). In addition, Compound C itself can increase the production of lipid ROS and Fe2^+^ (Fig. 6c and d). Therefore, it suggests that the balance of AMPK is critical for cell survival and the activation or inhibition of AMPK may induce stress response in cells in which ferroptosis may be involved. In short, these findings indicate that either metformin or AMPK imbalances can induce ferroptosis, and metformin may induce ferroptosis without the involvement of AMPK.

### The synergistic effect of SAS and metformin can effectively inhibit breast cancer cell

**Fig. 7:**
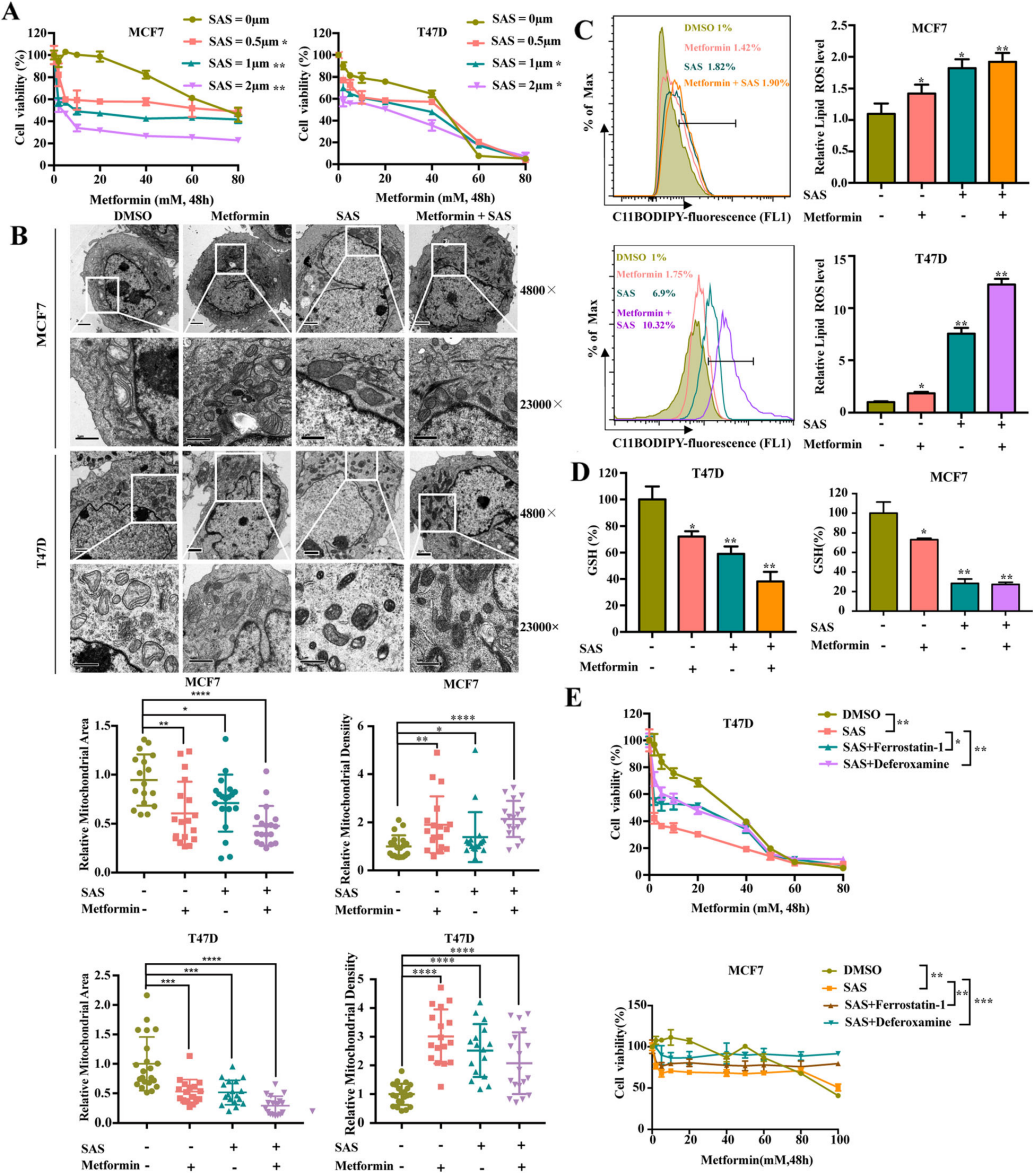
The synergistic effect of SAS and Metformin can effectively inhibit breast cancer. **A** T47D and MCF7 cells were treated with metformin (0–80 mM) in the absence or presence of sulfasalazine for 48 h. Cell viability was assayed. **B** Cell morphology was observed via transmission electron microscopy after cells were treated with metformin (5 mM) in the absence or presence of sulfasalazine for 48 h. The area and density of mitochondrial is quantitative analysis by using the ImageJ software. **C** T47D cells were treated with metformin (5 mM) in the absence or presence of sulfasalazine for 48 h. The relative lipid ROS levels were assayed using C11-BODIPY fluorescence. **D** T47D and MCF7 cells were treated with metformin (5 mM) in the absence or presence of sulfasalazine for 48 h. The relative levels of GSH were assayed. **E** T47D and MCF7 cells were treated with metformin and sulfasalazine in the absence or presence of ferrostatin-1 (1 μM) and Deferoxamine (20 μM) for 48 h. Cell viability was assayed

Our aforementioned data prompted us to examine whether inactivating SLC7A11 with sulfasalazine would potentiate metformin-induced lipid peroxidation and ferroptosis, thus sensitizing cancer cells to metformin. We treated cells with metformin combined with the SLC7A11 inhibitor sulfasalazine. Subsequently, we tested cell viability with a CCK8 assay to determine the synergistic effect of combining metformin and sulfasalazine. Our results showed that the combination of sulfasalazine and metformin can work in a synergistic manner to inhibit the proliferation of breast cancer, especially MDAMB231 cells (Fig. 7a, S5A). In addition, RSL3, a direct GPX4 inhibitor, also can work in synergy with metformin (Fig. S5D). Moreover, the combination of sulfasalazine and metformin further reduced the expression of SLC7A11 (Fig. S5B) and induced the reduction of mitochondrial volume and increased the membrane density (Fig. 7b and S5C). Furthermore, lipid peroxidation detection and GSH detection revealed that the combination of metformin and sulfasalazine significantly increased the lipid ROS level and inhibited GSH generation (Fig. 7c and d). DFO and Fer-1 effectively attenuated the killing effect of the metformin and sulfasalazine combination (Fig. 7e). Cumulatively, our data strongly suggests that the combination of sulfasalazine and metformin synergistically induces lipid peroxidation and ferroptosis and thus inhibits the proliferation of breast cancer.

### Sulfasalazine synergistically enhances the anti-cancer activity of metformin in vivo

**Fig. 8:**
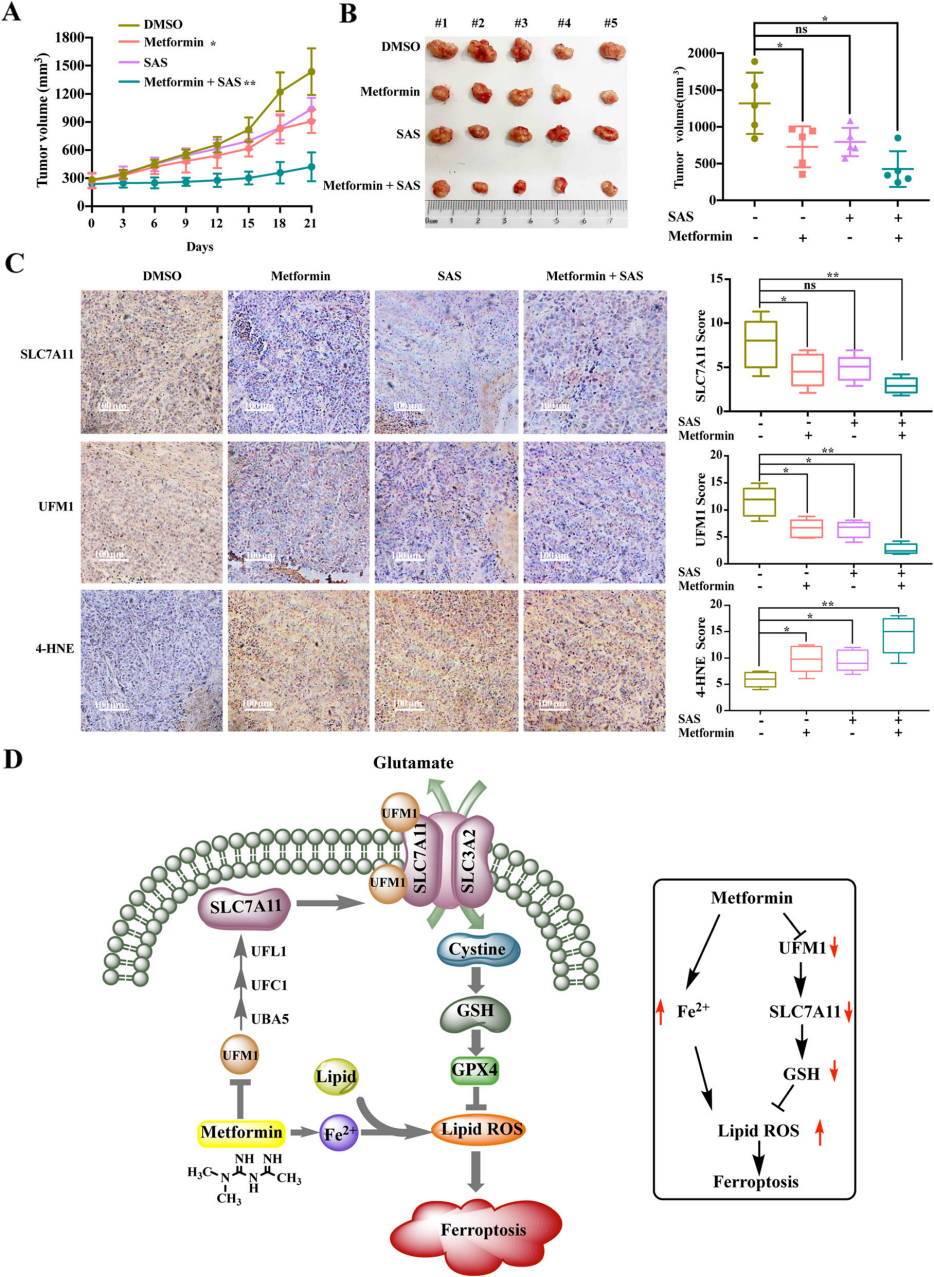
Sulfasalazine synergistically enhances the anti-cancer activity of Metformin in vivo. **A** Athymic nude mice were orthotopically injected with T47D cells and treated with metformin (200 mg/kg, three weeks) daily in drinking water with or without sulfasalazine administration via intraperitoneal injection until the end of the experiment. Tumor volumes are shown relative to the initial volume measured before treatment (*n* = 5 mice/group). **B** Representative isolated tumor images from each treatment group at day 21 after treatment are shown. **C** Representative images of immunohistochemical staining (SLC7A11, UFM1 and 4-HNE) of T47D cell-derived xenograft tumors with the indicated treatments. The box graphs show the quantification of IHC staining. Chi-square test was used for statistical analysis. **D** Schematic diagram showing metformin regulation of ferroptosis. SLC7A11 can be modified by UFM1; metformin exerts its anti-cancer effect by inhibiting UFMylation of SLC7A11 leading to ferroptosis in an AMPK-independent manner

We investigated whether Sulfasalazine enhances the anti-cancer activity of metformin in vivo. T47D cells were implanted subcutaneously into the right flank of immunodeficient nude mice. One week later, tumor-bearing mice were randomly divided into four groups and subsequently treated with DMSO, metformin, Sulfasalazine, or metformin with Sulfasalazine. Unsurprisingly, metformin and Sulfasalazine significantly suppressed tumor growth compared with the DMSO group (Fig. 8a and b). Among the four groups, the metformin and Sulfasalazine combination had the highest efficacy in inhibiting tumor growth (Fig. 8a and b). In addition, mice treated with metformin and Sulfasalazine showed lower UFM1 and SLC7A11 expression and higher 4-HNE expression, which have frequently been used as general markers of oxidative stress in tissues (Fig. 8c). These data indicate that Sulfasalazine sensitizes cancer cells to metformin-induced lipid peroxidation and ferroptosis, leading to tumor suppression in vivo.

## Discussion

In our current study, we demonstrated that metformin inhibited breast cancer mainly by inducing ferroptosis (Fig. 8d). Biochemically, metformin induced intracellular lipid ROS and Fe^2+^ accumulation and reduced the level of GSH (Fig. 1c and 2b, d). Morphologically, mitochondria become smaller with increased membrane density and significantly decreased volume after metformin administration (Fig. 2a). Mechanistically, metformin downregulated the expression of a key ferroptosis pathway protein, SLC7A11 (Fig. 3a). In addition, bioinformatics analysis and Western blotting showed that the ubiquitin-like molecule UFM1 is a target protein of metformin (Fig. 4a). Moreover, our results suggested that metformin could suppress UFM1 expression, subsequently inhibiting the overall cellular UFMylation level (Fig. 4b). Finally, we also found that SLC7A11 is a novel substrate for UFMylation modification by UFM1, which could be suppressed by metformin (Fig. 5e, g). In summary, our data suggest a novel anti-cancer mechanism of metformin in which metformin induces ferroptosis by repressing UFMylation of SLC7A11.

However, how metformin regulates UFM1 expression remains unclear. Our previous studies showed that low doses of metformin can downregulate the expression of long noncoding RNA H19, thereby inhibiting the metastasis of tumor cells. H19 can also bind to SAHH protein and affect its enzyme activity, consequently changing the binding state in the genomic DNA of methylated transferase DNMT3B and regulating genomic DNA methylation [44, 45]. Therefore, we speculated that metformin may regulate UFM1 via H19. Indeed, our ongoing research suggests that H19 is involved in the regulation of UFM1 (unpublished data). Moreover, in addition to SLC7A11, our study suggests that metformin can alter the overall cellular UFMylation modification level. Since UFMylation is extensively involved in various pathophysiological processes [21], whether metformin can exert other biological or therapeutic effects through UFM1 merits further studies.

In our study, we identified SLC7A11 as a new UFMylation substrate. However, an unanswered question is how UFM1 acts on SLC7A11. Currently, only a few UFM1 substrates have been found, including ASC1 [24] and DDRGK1 [25]. It is proposed that UFM1 primarily regulates target proteins by covalently binding to the substrate via lysine. Therefore, whether UFM1 acts on SLC7A11 in a similar lysine-related manner remains to be further studied. Exploring more substrates is helpful for further understanding of the functions of UFM1. In addition to system Xc^−^, ferroptosis is also regulated by other genes, including ACSL4 and FSP1 [37]. ACSL4 is responsible for shaping the cellular lipidome, which acts as an important node that determines the sensitivity or resistance of a cell to ferroptosis. In addition, the FSP1-catalyzed regeneration of CoQ10 (consuming NAD(P)H) exists as a stand-alone parallel anti-ferroptotic system, which cooperates with GPX4 and glutathione to suppress phospholipid peroxidation and ferroptosis [46]. However, whether and how UFM1 regulates these ferroptosis regulators is still not clear. It is also possible that additional mechanisms are involved in metformin-induced ferroptosis. Dissecting such mechanisms will remain an important area of future investigation.

The roles of AMPK in the anti-cancer effect of metformin have been well-established [1]. Accumulating evidence indicates an intimate link between AMPK and ferroptosis [41, 47, 48]; however, the results remain largely inconclusive. A recent study clearly showed that energy-stress-mediated AMPK activation likely inhibits ferroptosis [47]. Controversially, another study proposed that AMPK-mediated Beclin 1 (BECN1) phosphorylation promotes ferroptosis by inhibiting SLC7A11-mediated cystine transport [41]. Additionally, a recent study showed that mitochondria activation selectively promotes cystine-starvation-induced or erastin-induced ferroptosis, which suggests that mitochondria play an important role in regulating ferroptosis [48]. Our results suggest that either AMPK activation or inhibition can induce ferroptosis (Fig. 6a, b, c). Based on these findings, we speculate that the potential role of AMPK in ferroptosis is context-dependent and that metformin may induce ferroptosis without the involvement of AMPK. Interestingly, another study has shown that AICAR, an AMPK avtivator, can enhance the efficacy of rapamycin in human cancer cells [49]. The mTOR inhibitors including rapamycin can activate autophagy, and increasing evidences also prove that autophagy can regulate the development of ferroptosis [50]. Moreover, rapamycin has been applied in couple with erastin to enhance ferroptosis by inducing autophagy [51]. In our study, metformin can trigger ferroptosis but suppress autophagy (Fig. S2C, D). So, we suppose that metformin is similar to AICAR in that it also can combined with any mTOR inhibitors which may rescue the autophagy inhibited by metformin and thus further promote ferroptosis. There have also been studies showing that AMPK can regulate mTOR in a feedback loop manner [52]. In the absence of nutrients, AMPK is the metabolic checkpoint that inhibits cell growth which can be realized by suppressing the mTORC1 channel [53]. Some metabolic pathways, such as (selenium) thiol metabolism, fatty acid metabolism, iron handling, mevalonate pathway and mitochondrial respiration, directly impinge on the sensitivity of cells toward Lipid peroxidation and ferroptosis [54]. Therefore, studying the role of AMPK/mTOR-mediated metabolic pathway in ferroptosis will be a significant research direction in the future.

In addition, it’s interesting that cancer cells show a higher iron requirement than healthy cells which bring us to a major question that how do healthy primary cells react to the metformin treatment. Next, we tested the sensitivity of different breast cancer and normal breast cells to metformin (Fig. S1A, B.) The results showed that although metformin can effectively inhibit the proliferation of normal breast epithelial cells (HBL-100) and fibroblasts cells (NHFB), the toxicity of metformin to normal breast cells was similar to that of triple negative breast cancer (MDAMB231, BT549, HCC1937) and significantly lower than that of ER positive breast cancer cells including MCF7 and T47D. These results suggests that normal breast cells are relatively insensitive to metformin and the molecular subtype may affect the different effectivities of metformin treatment.

In this study, we elucidated how metformin regulates SLC7A11 and affects ferroptosis, but how metformin affects iron ions to regulate ferroptosis remains unclear. Iron-dependent lipid peroxidation is a hallmark of ferroptosis. Iron is an important component that composes a subunit of oxidase for lipid peroxidation [44, 55]. Iron not only directly catalyzes the formation of ROS but is also involved in the synthesis of lipoxygenases (LOXs) that oxidize cellular membrane polyunsaturated fatty acids (PUFAs), resulting in lipid peroxides in ferroptosis. Hence, iron is an essential component of ferroptosis [56]. Regulation of iron metabolism and ferritinophagy are additional potential points in control of ferroptosis. Some studies have shown that metformin can regulate iron ion homeostasis, but the specific mechanism is unknown. One study showed that metformin can modulate ferritin heavy chain (FHC) against the oxidative stress induced by doxorubicin [57]. Since iron can be consumed and stored by mitochondria [58], there may be a mutually regulatory relationship between mitochondria and iron ions that affect the development of ferroptosis. In addition, our data show that metformin can cause mitochondrial membrane potential disorder, suggesting that metformin may regulate iron ion levels through mitochondria. The regulatory effects of metformin in other iron metabolism signaling pathways still need to be further studied.

The anti-cancer effect of metformin has been closely correlated with apoptosis, autophagy, and other regulated cell death pathways [7, 59]. To the best of our knowledge, our study is the first to reveal that metformin can exert an anti-cancer effect by inducing ferroptosis. There is a growing body of evidence suggesting the presence of crosstalk between ferroptosis and other regulated cell death types. Autophagy plays an important role in the process of ferroptosis by regulating cell iron homeostasis and cell ROS production, and in contrast, ferroptosis induction also activates autophagy [50]. Another study demonstrated that autophagy contributes to ferroptosis via degradation of ferritin [60]. Our results showed that metformin induced ferroptosis (Fig. 2) and apoptosis (Fig. S2A/B) and inhibited autophagy (Fig. S2C). Besides, only ferroptosis inhibitors could significantly inhibit the anti-cancer effect of metformin, while apoptosis, autophagy, and necrosis inhibitors had only a slight and insignificant effect (Fig. 1a). Based on our data, we propose that the cancer cell suppression effect of metformin is mainly due to ferroptosis induction. However, we have not investigated whether metformin may contribute to other forms of death by inducing ferroptosis. Due to the complexity of the crosstalk among the different regulated cell death types, future studies are guaranteed.

Previous studies have suggested an association between metformin use and improved outcome in patients with diabetes and breast cancer, indicating that metformin has great potential in the treatment of breast cancer [61, 62]. However, another study demonstrated that the addition of metformin to first-line chemotherapy in metastatic breast cancer did not provide a meaningful clinical benefit in terms of PFS or OS [63]. It is critical to screen the appropriate molecular targets and precisely identify patients who have the highest likelihood of benefiting from metformin. We found the SLC7A11 expression was significantly negatively correlated with the prognosis of patients with breast cancer (Fig. 5b). Overall, we propose that breast cancer cases with higher SLC7A11 levels might be appropriate candidates for Breast Cancer treatment, breaking down the mechanisms underlying the protection of SLC7A11 in some breast cancer cells may be a potential avenue for future research in the treatment of breast cancer.

Sulfasalazine is a Food and Drug Administration-approved drug commonly used for the treatment of rheumatoid arthritis. It is a well-characterized specific inhibitor of SLC7A11-mediated cystine transport and has been shown to inhibit the growth, invasion, and metastasis of several cancer types [18, 64]. Our data showed that sulfasalazine synergistically increased the level of ferroptosis and enhanced the anti-cancer effect of metformin in vitro and in vivo (Fig. 7a-e and 8a, b), but whether metformin in combination with SAS induces other forms of cell death is still not clear, and remains to be explored. Notably, we also found that combining Sulfasalazine with metformin effectively suppressed the relatively “metformin resistant” MDAMB231 cancer cells (Fig. S5A). Consistent with our findings, a recent study demonstrated that metformin combined with hemin is effective for breast cancer [65]. Thus, screening a new metformin-based drug combination might be a promising strategy to further enhance the anti-cancer efficacy of metformin.

## Conclusions

In summary, to the best of our knowledge, this study is the first to establish a link between metformin and ferroptosis. Thus, to fully harness the potential of metformin for cancer treatment, it will be important in the future work to determine not only cellular determinants of ferroptosis sensitivity and resistance but also systemic responses and the mechanisms through which these responses are interlinked with other types of regulated cell death. Finally, we envision that a detailed understanding of the exact genetic or tumor contexts that maximize treatment efficacy will likely govern the therapeutic utility of metformin.

## Supplementary Information

**Additional file 1: Figure S1.** Metformin inhibits breast cancer cell proliferation, induces an increase in the lipid ROS level and decreases the GSH level

**Additional file 2: Figure S2.** Metformin promotes apoptosis, inhibits autophagy and induces mitochondrial membrane potential disorder

**Additional file 3: Figure S3.** SLC7A11 and UFM1 were involved in the Ferroptosis process

**Additional file 4: Figure S4.** SLC7A11 and UFM1 did not affect cell proliferation by themselves

**Additional file 5: Figure S5.** The synergistic effect of SAS and Metformin can effectively inhibit breast cancer

## Data Availability

The datasets used and/or analyzed during the current study are available from the corresponding author on reasonable request.

## Abbreviations

ROS: Reactive oxygen species
AMPK: AMP-activated protein kinase
SAS: Sulfasalazine
DFO: Deferoxamine mesylate
Fer-1: Ferrostatin-1
Necro: E-necrosulfonamide
CHX: Cycloheximide
3-MA: 3-Methyladenine
4-HNE: 4 Hydroxynonenal

## References

[1] Pernicova I, Korbonits M (2014). Metformin-mode of action and clinical implications for diabetes and cancer. Nat Rev Endocrinol.

[2] Barzilai N, Crandall JP, Kritchevsky SB, Espeland MA (2016). Metformin as a tool to target aging. Cell Metab.

[3] Soberanes S, Misharin AV, Jairaman A, Morales-Nebreda L, McQuattie-Pimentel AC, Cho T (2019). Metformin targets mitochondrial electron transport to reduce air-pollution-induced thrombosis. Cell Metabol.

[4] Zhang CS, Li M, Ma T, Zong Y, Cui J, Feng JW, Wu YQ, Lin SY, Lin SC (2016). Metformin activates AMPK through the lysosomal pathway. Cell Metab.

[5] Stynen B, Abd-Rabbo D, Kowarzyk J, Miller-Fleming L, Aulakh SK, Garneau P, Ralser M, Michnick SW (2018). Changes of cell biochemical states are revealed in protein homomeric complex dynamics. Cell.

[6] Queiroz EAIF, Puukila S, Eichler R, Sampaio SC, Forsyth HL, Lees SJ (2014). Metformin induces apoptosis and cell cycle arrest mediated by oxidative stress, AMPK and FOXO3a in MCF-7 breast cancer cells. PLoS ONE.

[7] Xiao Z, Gaertner S, Morresi-Hauf A, Genzel R, Duell T, Ullrich A, Knyazev PG (2017). Metformin triggers autophagy to attenuate drug-induced apoptosis in NSCLC cells, with minor effects on tumors of diabetic patients. Neoplasia.

[8] Hassannia B, Vandenabeele P, Vanden BT (2019). Targeting Ferroptosis to Iron out Cancer. Cancer Cell.

[9] Xie Y, Hou W, Song X, Yu Y, Huang J, Sun X, Kang R, Tang D (2016). Ferroptosis: process and function. Cell Death Differ.

[10] Mancias JD, Wang X, Gygi SP, Harper JW, Kimmelman AC (2014). Quantitative proteomics identifies NCOA4 as the cargo receptor mediating ferritinophagy. Nature.

[11] Richardson DR, Lane DJ, Becker EM, Huang ML, Whitnall M, Rahmanto YS (2010). Mitochondrial iron trafficking and the integration of iron metabolism between the mitochondrion and cytosol. Proc Natl Acad Sci U S A.

[12] Lill R, Srinivasan V, Mühlenhoff U (2014). The role of mitochondria in cytosolic-nuclear iron-sulfur protein biogenesis and in cellular iron regulation. Curr Opin Microbiol.

[13] Yang WS, Stockwell BR (2016). Ferroptosis: death by lipid peroxidation. Trends Cell Biol.

[14] Stockwell BR, Friedmann Angeli JP, Bayir H, Bush AI, Conrad M, Dixon SJ (2017). Ferroptosis: a regulated cell death nexus linking metabolism, redox biology, and disease. Cell.

[15] Conrad M, Kagan VE, Bayir H, Pagnussat GC, Head B, Traber MG, Stockwell BR (2018). Regulation of lipid peroxidation and ferroptosis in diverse species. Genes Dev.

[16] Koppula P, Zhang Y, Zhuang L, Gan B (2018). Amino acid transporter SLC7A11/xCT at the crossroads of regulating redox homeostasis and nutrient dependency of cancer. Cancer Commun.

[17] Dixon SJ, Lemberg KM, Lamprecht MR, Skouta R, Zaitsev EM, Gleason CE, Patel DN, Bauer AJ, Cantley AM, Yang WS, Morrison B, Stockwell BR (2012). Ferroptosis: an iron-dependent form of nonapoptotic cell death. Cell.

[18] Gout PW, Buckley AR, Simms CR, Bruchovsky N (2001). Sulfasalazine, a potent suppressor of lymphoma growth by inhibition of the xc? cystine transporter: a new action for an old drug. Leukemia.

[19] Lei G, Zhang Y, Koppula P, Liu X, Zhang J, Lin SH, Ajani JA, Xiao Q, Liao Z, Wang H, Gan B (2020). The role of ferroptosis in ionizing radiation-induced cell death and tumor suppression. Cell Res.

[20] Komatsu M, Chiba T, Tatsumi K, Lemura SI, Tanida I, Okazaki N (2004). A novel protein-conjugating system for Ufm1, a ubiquitin-fold modifier. EMBO J.

[21] Daniel J, Liebau E (2014). The Ufm1 Cascade. Cells.

[22] Tatsumi K, Sou YS, Tada N, Nakamura E, Iemura SI, Natsume T, Kang SH, Chung CH, Kasahara M, Kominami E, Yamamoto M, Tanaka K, Komatsu M (2010). A novel type of E3 ligase for the Ufm1 conjugation system. J Biol Chem.

[23] Tatsumi K, Yamamoto-Mukai H, Shimizu R, Waguri S, Sou YS, Sakamoto A, Taya C, Shitara H, Hara T, Chung CH, Tanaka K, Yamamoto M, Komatsu M (2011). The Ufm1-activating enzyme Uba5 is indispensable for erythroid differentiation in mice. Nat Commun.

[24] Yoo HM, Kang SH, Kim JY, Lee JE, Seong MW, Lee SW, Ka SH, Sou YS, Komatsu M, Tanaka K, Lee ST, Noh DY, Baek SH, Jeon YJ, Chung CH (2014). Modification of ASC1 by UFM1 is crucial for ERα transactivation and breast Cancer development. Mol Cell.

[25] Cai Y, Pi W, Sivaprakasam S, Zhu X, Zhang M, Chen J, Makala L, Lu C, Wu J, Teng Y, Pace B, Tuan D, Singh N, Li H (2015). UFBP1, a key component of the Ufm1 conjugation system, is essential for Ufmylation-mediated regulation of erythroid development. PLoS Genet.

[26] Lemaire K, Rodrigo M, Granvik M, Hohmeier H, Hendrickx N, Newgard C (2010). New players in the beta cell ER stress response: UFM1 and UFBP1. Diabetologia.

[27] Jiang L, Ning K, Li T, Wang SJ, Su T, Hibshoosh H (2015). Ferroptosis as a p53-mediated activity during tumour suppression. Nature.

[28] Zhang Y, Shi J, Liu X, Feng L, Gong Z, Koppula P, Sirohi K, Li X, Wei Y, Lee H, Zhuang L, Chen G, Xiao ZD, Hung MC, Chen J, Huang P, Li W, Gan B (2018). BAP1 links metabolic regulation of ferroptosis to tumour suppression. Nat Cell Biol.

[29] Fan Z, Wirth AK, Chen D, Wruck CJ, Rauh M, Buchfelder M, Savaskan N (2017). Nrf2-keap1 pathway promotes cell proliferation and diminishes ferroptosis. Oncogenesis.

[30] Mukhopadhyay S, Goswami D, Adiseshaiah PP, Burgan W, Yi M, Guerin TM, Kozlov SV, Nissley DV, McCormick F (2020). Undermining glutaminolysis bolsters chemotherapy while NRF2 promotes chemoresistance in KRAS-driven pancreatic cancers. Cancer Res.

[31] Mukhopadhyay S, Heiden MG, Mccormick F (2021). The metabolic landscape of RAS-driven cancers from biology to therapy. Nat Cancer.

[32] Li P, Zhao M, Parris AB, Feng X, Yang X (2015). P53 is required for metformin-induced growth inhibition, senescence and apoptosis in breast cancer cells. Biochem Biophys Res Commun.

[33] Urpilainen E, Kangaskokko J, Puistola U, Karihtala P (2019). Metformin diminishes the unfavourable impact of Nrf2 in breast cancer patients with type 2 diabetes. Tumor Biol.

[34] Schulten HJ (2018). Pleiotropic effects of metformin on cancer. Int J Mol Sci.

[35] Fan C, Wang Y, Liu Z, Sun Y, Wang X, Wei G (2015). Metformin exerts anticancer effects through the inhibition of the sonic hedgehog signaling pathway in breast cancer. Int J Mol Med.

[36] Rothman RJ, Serroni A, Farber JL (1992). Cellular pool of transient ferric iron, chelatable by deferoxamine and distinct from ferritin, that is involved in oxidative cell injury. Mol Pharmacol.

[37] Stockwell BR, Jiang X, Gu W (2020). Emerging mechanisms and disease relevance of Ferroptosis. Trends Cell Biol.

[38] Chen L, Hambright WS, Na R, Ran Q (2015). Ablation of the ferroptosis inhibitor glutathione peroxidase 4 in neurons results in rapid motor neuron degeneration and paralysis. J Biol Chem.

[39] Yang WS, Sriramaratnam R, Welsch ME, Shimada K, Skouta R, Viswanathan VS (2014). Regulation of Ferroptotic Cancer cell death by GPX4. Cell.

[40] Angeli JPF, Shah R, Pratt DA, Conrad M (2017). Ferroptosis inhibition: mechanisms and opportunities. Trends Pharmacol Sci.

[41] Song X, Zhu S, Chen P, Hou W, Wen Q, Liu J, et al. AMPK-Mediated BECN1 Phosphorylation Promotes Ferroptosis by Directly Blocking System Xc– Activity. Current Biol. 2018;28:2388–99. 10.1016/j.cub.2018.05.094. 10.1016/j.cub.2018.05.094 PMC6081251 30057310

[42] Cao JY, Dixon SJ (2016). Mechanisms of ferroptosis. Cell Mol Life Sci.

[43] Ota S, Horigome K, Ishii T, Nakai M, Hayashi K, Kawamura T, Kishino A, Taiji M, Kimura T (2009). Metformin suppresses glucose-6-phosphatase expression by a complex I inhibition and AMPK activation-independent mechanism. Biochem Biophys Res Commun.

[44] Zhong T, Men Y, Lu L, Geng T, Zhou J, Mitsuhashi A, Shozu M, Maihle NJ, Carmichael GG, Taylor HS, Huang Y (2017). Metformin alters DNA methylation genome-wide via the H19/SAHH axis. Oncogene.

[45] Zhou J, Yang L, Zhong T, Mueller M, Men Y, Zhang N, Xie J, Giang K, Chung H, Sun X, Lu L, Carmichael GG, Taylor HS, Huang Y (2015). H19 lncRNA alters DNA methylation genome wide by regulating S-adenosylhomocysteine hydrolase. Nat Commun.

[46] Doll S, Freitas FP, Shah R, Aldrovandi M, da Silva MC, Ingold I, Goya Grocin A, Xavier da Silva TN, Panzilius E, Scheel CH, Mourão A, Buday K, Sato M, Wanninger J, Vignane T, Mohana V, Rehberg M, Flatley A, Schepers A, Kurz A, White D, Sauer M, Sattler M, Tate EW, Schmitz W, Schulze A, O’Donnell V, Proneth B, Popowicz GM, Pratt DA, Angeli JPF, Conrad M (2019). FSP1 is a glutathione-independent ferroptosis suppressor. Nature.

[47] Lee H, Zandkarimi F, Zhang Y, Meena JK, Kim J, Zhuang L, Tyagi S, Ma L, Westbrook TF, Steinberg GR, Nakada D, Stockwell BR, Gan B (2020). Energy-stress-mediated AMPK activation inhibits ferroptosis. Nat Cell Biol.

[48] Gao M, Yi J, Zhu J, Minikes AM, Monian P, Thompson CB (2019). Role of Mitochondria in Ferroptosis. Mol Cell.

[49] Mukhopadhyay S, Chatterjee A, Kogan D, Patel D, Foster DA (2015). 5-aminoimidazole-4-carboxamide-1-β-4-ribofuranoside (AICAR) enhances the efficacy of rapamycin in human cancer cells. Cell Cycle.

[50] Gao M, Monian P, Pan Q, Zhang W, Xiang J, Jiang X (2016). Ferroptosis is an autophagic cell death process. Cell Res.

[51] Li Y, Wang X, Yan J, Liu Y, Yang R, Pan D, Wang L, Xu Y, Li X, Yang M (2019). Nanoparticle ferritin-bound erastin and rapamycin: a nanodrug combining autophagy and ferroptosis for anticancer therapy. Biomater Sci.

[52] Mukhopadhyay S, Saqcena M, Chatterjee A, Garcia A, Frias MA, Foster DA (2015). Reciprocal regulation of AMP-activated protein kinase and phospholipase D. J Biol Chem.

[53] Mukhopadhyay S, Saqcena M, Foster DA (2015). Synthetic lethality in KRas-driven cancer cells created by glutamine deprivation. Oncoscience.

[54] Zheng J, Conrad M (2020). The metabolic underpinnings of Ferroptosis. Cell Metab.

[55] Zhou B, Liu J, Kang R, Klionsky DJ, Kroemer G, Tang D (2020). Ferroptosis is a type of autophagy-dependent cell death. Semin Cancer Biol.

[56] Stoyanovsky DA, Tyurina YY, Shrivastava I, Bahar I, Tyurin VA, Protchenko O, Jadhav S, Bolevich SB, Kozlov AV, Vladimirov YA, Shvedova AA, Philpott CC, Bayir H, Kagan VE (2019). Iron catalysis of lipid peroxidation in ferroptosis: regulated enzymatic or random free radical reaction?. Free Radic Biol Med.

[57] Asensio-López MC, Sánchez-Más J, Pascual-Figal DA, Abenza S, Pérez-Martínez MT, Valdés M, Lax A (2013). Involvement of ferritin heavy chain in the preventive effect of metformin against doxorubicin-induced cardiotoxicity. Free Radic Biol Med.

[58] Lane DJR, Merlot AM, Huang MLH, Bae DH, Jansson PJ, Sahni S, Kalinowski DS, Richardson DR (2015). Cellular iron uptake, trafficking and metabolism: key molecules and mechanisms and their roles in disease. Biochim et Biophys Acta Mol Cell Res.

[59] Buzzai M, Jones RG, Amaravadi RK, Lum JJ, DeBerardinis RJ, Zhao F (2007). Systemic treatment with the antidiabetic drug metformin selectively impairs p53-deficient tumor cell growth. Cancer Res.

[60] Hou W, Xie Y, Song X, Sun X, Lotze MT, Zeh HJ (2016). Autophagy promotes ferroptosis by degradation of ferritin. Autophagy.

[61] Jiralerspong S, Palla SL, Giordano SH, Meric-Bernstam F, Liedtke C, Barnett CM, Hsu L, Hung MC, Hortobagyi GN, Gonzalez-Angulo AM (2009). Metformin and pathologic complete responses to neoadjuvant chemotherapy in diabetic patients with breast cancer. J Clin Oncol.

[62] Sonnenblick A, Agbor-Tarh D, Bradbury I, Di Cosimo S, Azim HA, Fumagalli D (2017). Impact of diabetes, insulin, and metformin use on the outcome of patients with human epidermal growth factor receptor 2–positive primary breast Cancer: analysis from the ALTTO phase III randomized trial. J Clin Oncol.

[63] Nanni O, Amadori D, De Censi A, Rocca A, Freschi A, Bologna A (2019). Metformin plus chemotherapy versus chemotherapy alone in the first-line treatment of HER2-negative metastatic breast cancer. The MYME randomized, phase 2 clinical trial. Breast Cancer Res Treat.

[64] Chen RS, Song YM, Zhou ZY, Tong T, Li Y, Fu M, Guo XL, Dong LJ, He X, Qiao HX, Zhan QM, Li W (2009). Disruption of xCT inhibits cancer cell metastasis via the caveolin-1/β-catenin pathway. Oncogene.

[65] Lee J, Yesilkanal AE, Wynne JP, Frankenberger C, Liu J, Yan J (2019). Effective breast cancer combination therapy targeting BACH1 and mitochondrial metabolism. Nature.

