# Supplementary figures and images for: Metformin induces Ferroptosis by inhibiting UFMylation of SLC7A11 in breast cancer
Source: J Exp Clin Cancer Res. 2021 Jun 23;40:206. doi: 10.1186/s13046-021-02012-7 (PMC8223374; doi:10.1186/s13046-021-02012-7)

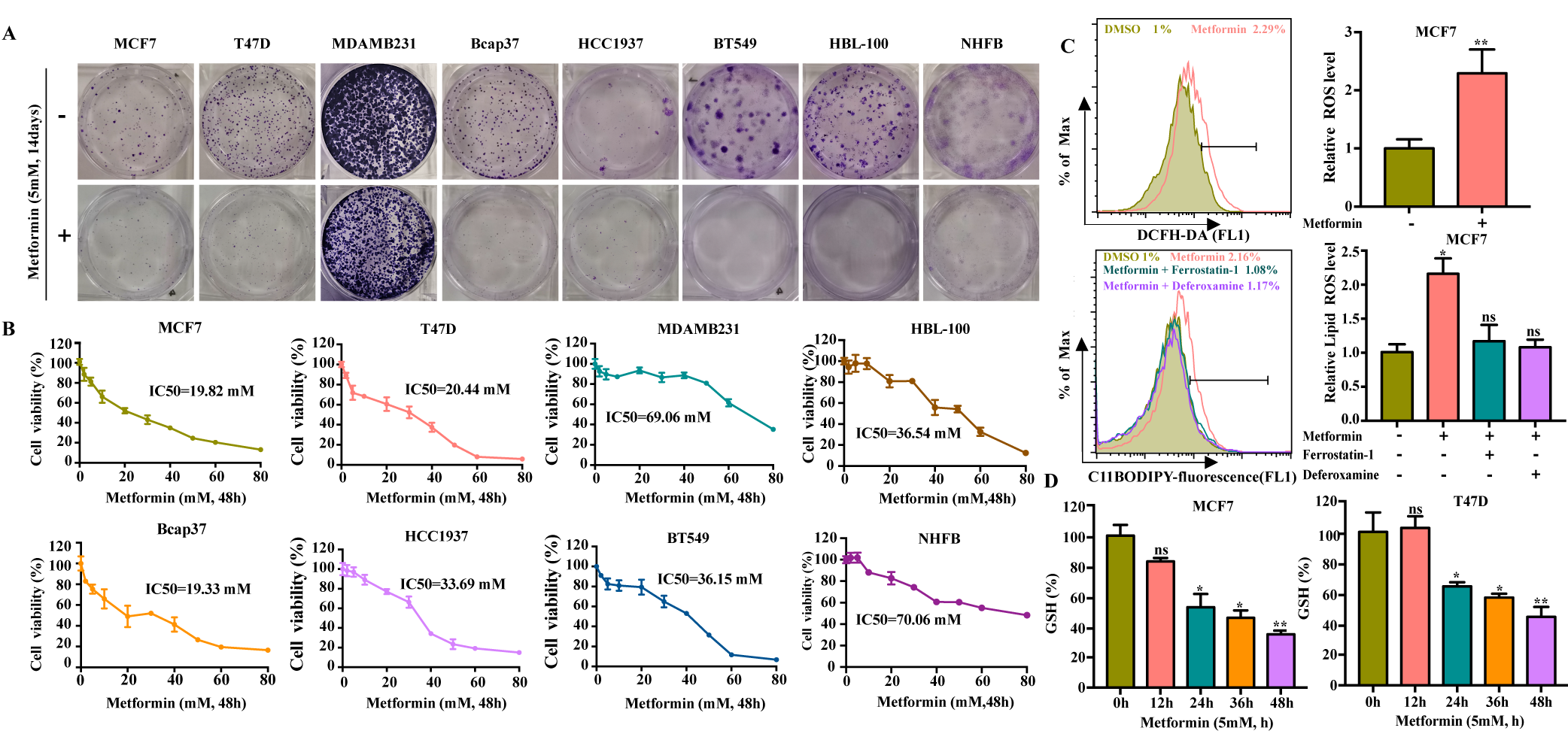

Supplement: Supplementary file 1 — Additional file 1: Figure S1. Metformin inhibits breast cancer cell proliferation, induces an increase in the lipid ROS level and decreases the GSH level [file 13046_2021_2012_MOESM1_ESM.tif]

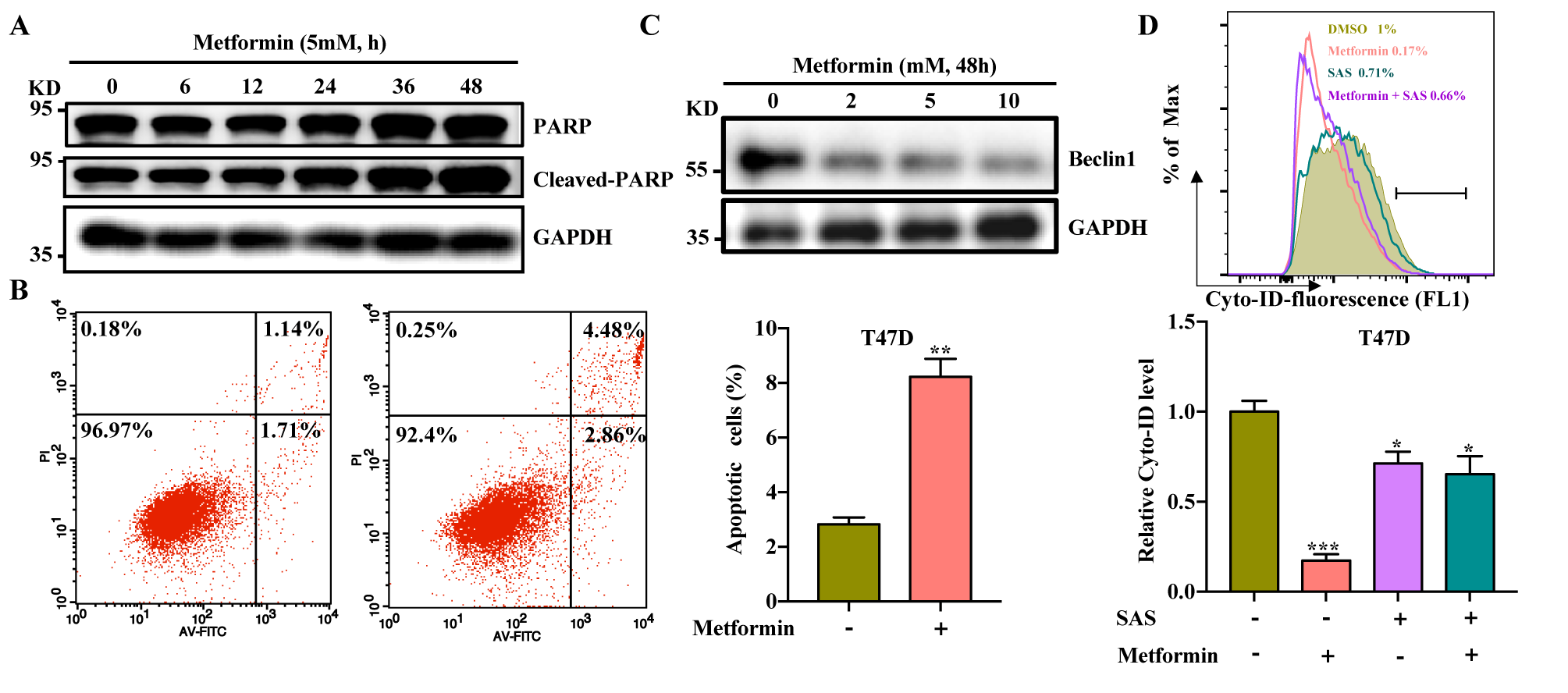

Supplement: Supplementary file 2 — Additional file 2: Figure S2. Metformin promotes apoptosis, inhibits autophagy and induces mitochondrial membrane potential disorder [file 13046_2021_2012_MOESM2_ESM.tif]

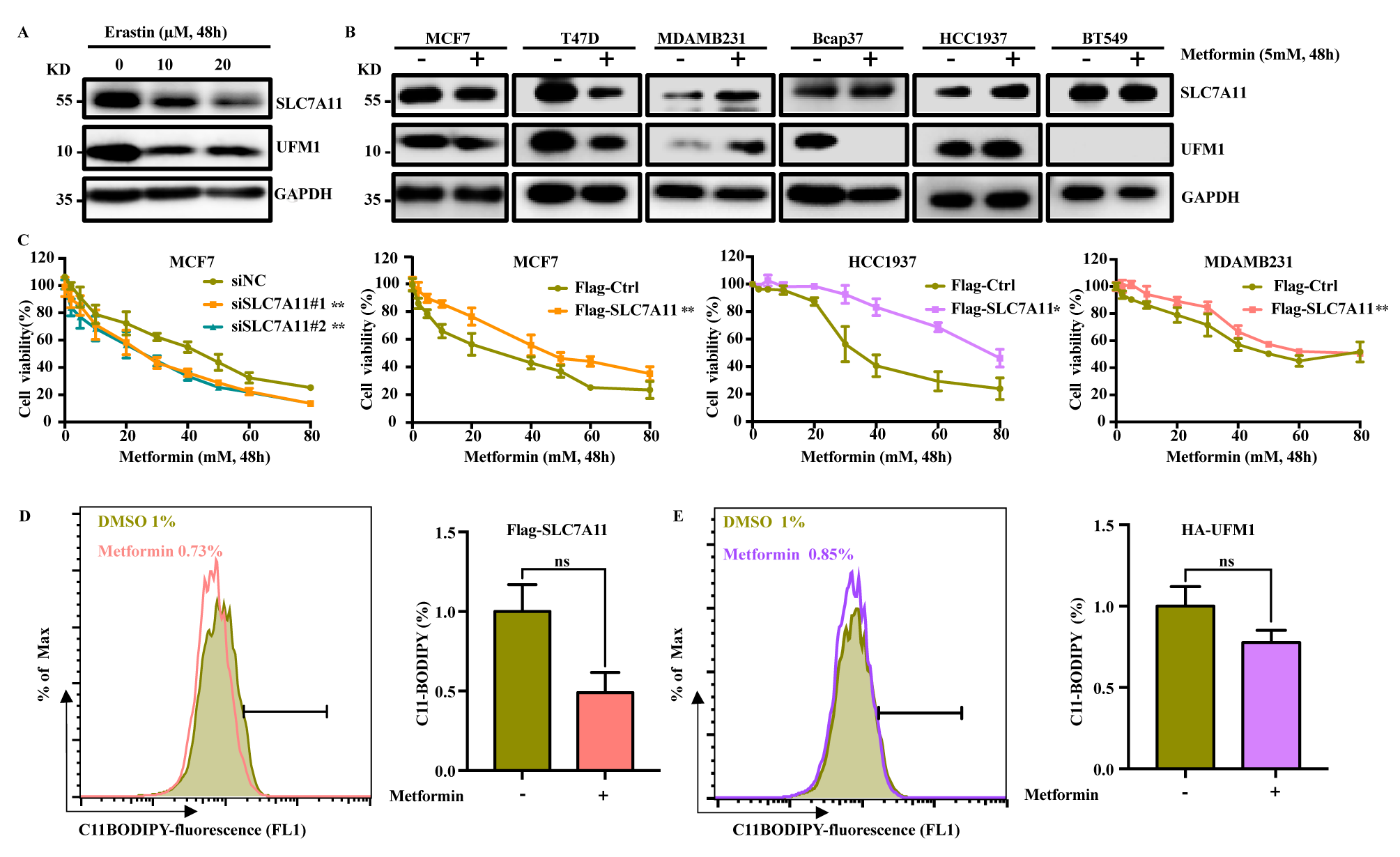

Supplement: Supplementary file 3 — Additional file 3: Figure S3. SLC7A11 and UFM1 were involved in the Ferroptosis process [file 13046_2021_2012_MOESM3_ESM.tif]

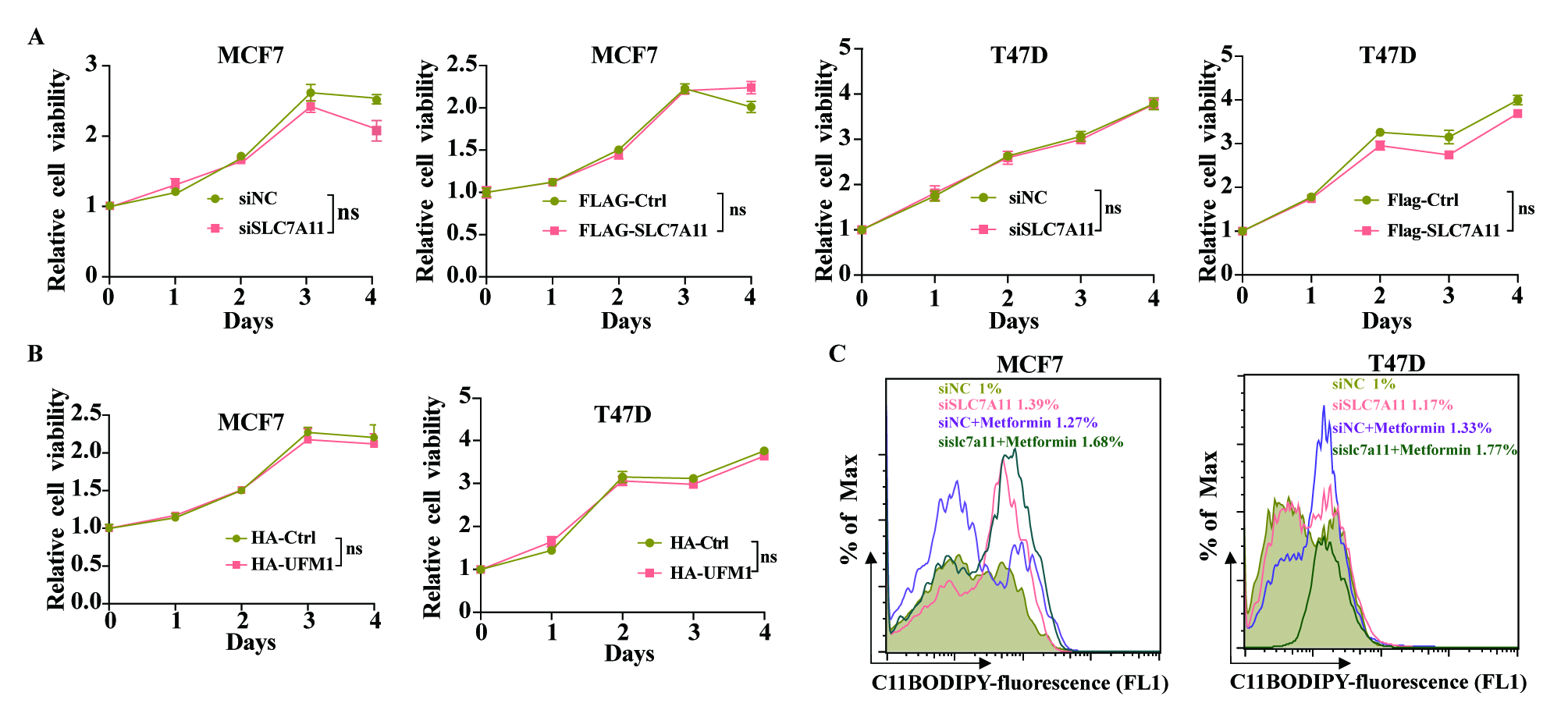

Supplement: Supplementary file 4 — Additional file 4: Figure S4. SLC7A11 and UFM1 did not affect cell proliferation by themselves [file 13046_2021_2012_MOESM4_ESM.tif]

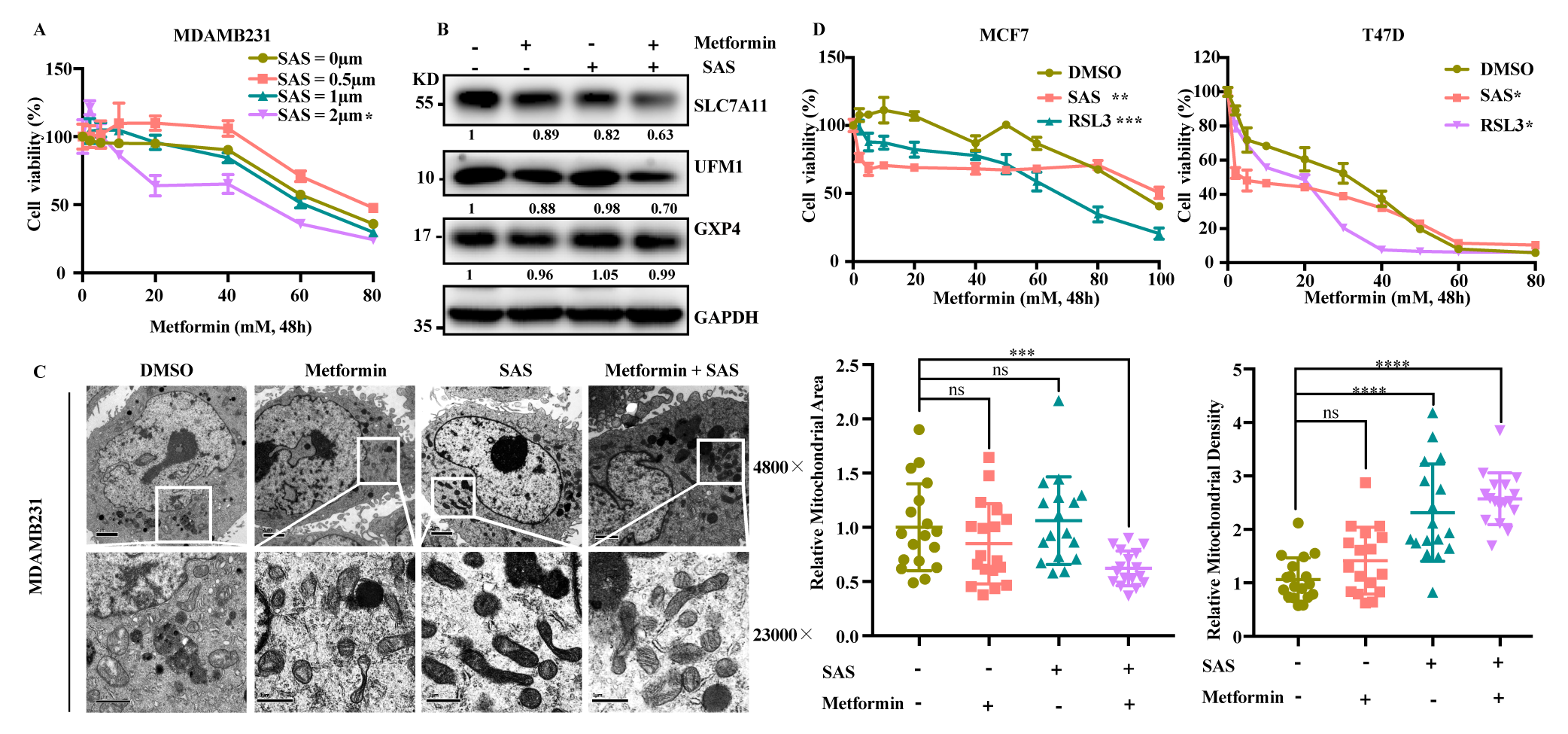

Supplement: Supplementary file 5 — Additional file 5: Figure S5. The synergistic effect of SAS and Metformin can effectively inhibit breast cancer [file 13046_2021_2012_MOESM5_ESM.tif]
